# Comparison of the lipidomic signature of fatty liver in children and adults: a cross-sectional study

**DOI:** 10.1097/MPG.0000000000003418

**Published:** 2022-02-19

**Authors:** Jake P. Mann, Benjamin Jenkins, Samuel Furse, Stuart G. Snowden, Anna Alisi, Laura G. Draijer, EU-PNAFLD investigators, Kylie Karnebeek, Deirdre A. Kelly, Bart G. Koot, Antonella Mosca, Camilla Salvestrini, Indra van Mourik, Anita Vreugdenhil, Matthias Zilbauer, Albert Koulman

**Affiliations:** 1 Institute of Metabolic Science, University of Cambridge, Cambridge, UK; 2 Core Metabolomics and Lipidomics Laboratory, Wellcome Trust-MRC Institute of Metabolic Science, University of Cambridge; 3 Department of Biological Sciences, Royal Holloway University of London, Egham, UK; 4 Research Unit of Molecular Genetics of Complex Phenotypes, Bambino Gesù Children’s Hospital, IRCCS, Rome, Italy; 5 Department of Paediatric Gastroenterology and Nutrition, Amsterdam University Medical Centers, Emma Children’s Hospital, Amsterdam, The Netherlands; 6 Centre for Overweight Adolescent and Children’s Healthcare (COACH), Department of pediatrics, School of Nutrition and Translational Research in Metabolism (NUTRIM), Maastricht University Medical Centre, The Netherlands; 7 Liver Unit, Birmingham Women’s & Children’s Hospital, and University of Birmingham, Birmingham, UK; 8 Hepatology Gastroenterology and Nutrition, Bambino Gesù Children’s Hospital, Rome, Italy; 9 Department of Paediatric Gastroenterology and Nutrition, Addenbrooke’s Hospital, Cambridge, UK; 10 Department of Paediatrics, University of Cambridge, UK

**Keywords:** hepatic steatosis, biomarker, fibrosis, diabetes

## Abstract

**Objective:**

Non-alcoholic fatty liver disease (NAFLD) is an increasingly common condition in children characterized by insulin resistance and altered lipid metabolism. Affected patients are at increased risk of cardiovascular disease (CVD) and children with NAFLD are likely to be at risk of premature cardiac events. Evaluation of the plasma lipid profile of children with NAFLD offers the opportunity to investigate these perturbations and understand how closely they mimic the changes seen in adults with cardiometabolic disease.

**Methods:**

We performed untargeted liquid chromatography mass spectrometry (LC-MS) plasma lipidomics on 287 children: 19 lean controls, 146 from an obese cohort, and 122 NAFLD cases who had undergone liver biopsy. Associations between lipid species and liver histology were assessed using regression adjusted for age and sex. Results were then replicated using data from 9,500 adults with metabolic phenotyping.

**Results:**

More severe paediatric NAFLD was associated with lower levels of long chain, polyunsaturated phosphatidylcholines (PC) and triglycerides (TG). Similar trends in PC and TG chain length and saturation were seen in adults with hepatic steatosis. However, many of the specific lipids associated with NAFLD differed between children and adults. Five lipids replicated in adults (including PC(36:4)) have been directly linked to death and cardiometabolic disease, as well as indirectly via genetic variants.

**Conclusion:**

These findings suggest that, whilst similar pathways of lipid metabolism are perturbed in paediatric NAFLD as in cardiometabolic disease in adults, the specific lipid signature in children is different.

## Introduction

Non-alcoholic fatty liver disease (NAFLD) is a common, chronic disorder that is closely linked to obesity and insulin resistance^(1, 2)^. Most of the morbidity and mortality in patients with NAFLD occurs due to complications of cardiovascular disease though a proportion develop cirrhosis^(3)^. Individuals with a higher fibrosis stage^(4)^ or more active inflammation (non-alcoholic steatohepatitis (NASH))^(5)^ are at increased risk of end-stage liver disease and cardiovascular disease (CVD)^(6)^.

Whilst the long-term outcomes of NAFLD in children have not yet been formally established with the same degree of confidence as in adults^(7, 8)^, they are believed to be similar, including risk of CVD. However, paediatric NAFLD has several unique features, including prominent peri-portal inflammation^(9)^. Therefore, it is not entirely clear to what extent paediatric and adult NAFLD differ.

Lipidomics is a technique that aims to measure the concentration of hundreds of lipid species. It has been used by several groups to gain insight into altered lipid metabolism in NAFLD. Liver samples^(10–12)^, venous-^(13, 14)^ and portal-blood^(15)^ have been studied, showing specific lipid species to associate with histological severity of NAFLD in adults. This work has identified perturbation of pathways including increased hepatic *de novo* lipogenesis (DNL), desaturase activity, and phospholipid metabolism. To date, lipidomic studies in children have focused differentiating NAFLD patients from healthy or obese controls^(16, 17)^. However, none of these studies have included histologically characterized cases. Therefore it is not clear whether the observed changes are reflective of underlying insulin resistance or specific to NAFLD.

In this study we used plasma lipidomics to investigate lipid metabolism in children with NAFLD. Specifically, we aimed to: (i) identify lipids associated with the histological severity of NAFLD; (ii) determine if similar changes were observed in separate cohort of obese children; (iii) identify any overlap in a cohort of adults with NAFLD; (iv) and to explore the potential significance of these lipids on cardiometabolic disease outcomes using data from adults. We hypothesized that the lipid signature of paediatric NAFLD would be largely reflective of insulin resistance and therefore would be associated with cardiometabolic disease in adults.

## Methods

### Participants

An overview of the study design is shown in Figure, Supplemental Digital Content 1. Three groups of participants were included in this cross-sectional study: lean controls, a cohort of children who were overweight or obese (‘obesity cohort’), and cases with suspected advanced NAFLD who had undergone liver biopsy (‘biopsied NAFLD cases’). In addition, we used publicly available data from adults cohorts^(18–20)^. All participants (or their parents) gave written informed consent and were recruited between 2014-2019, for the below ethically-approved studies, which were confirmed with the Declaration of Helsinki principles.

Lean controls were recruited as part of the Translational Research in Intestinal Physiology and Pathology (TRIPP) Study at Cambridge University (UK), which was approved by East of England - Cambridge South Research Ethics Committee (REC 17/EE/0265). These children had been referred due to diarrhea, vomiting, or abdominal pain and underwent endoscopy to rule out gastrointestinal disease. They were found to have no evidence of pathology after thorough assessment and had complete resolution of any symptoms. There is a low likelihood of NAFLD is this control group who were lean (body mass index (BMI) z-score <1.04) and had normal liver biochemistry.

The obesity cohort was recruited from paediatric obesity clinics at Maastricht Children’s Hospital (under ethical approval METC 13-4-130) and Amsterdam University Medical Centers (under ethical approvals MEC 2017_306 and MEC 07/141). Children were referred to these clinics from their primary care physicians due to being overweight or obese and were then subsequently investigated for co-morbidities (and secondary causes of obesity). As there was no clinical indication for liver biopsy, it was not possible to conclusively identify or exclude NAFLD in all children from the obesity cohort. However, a subset underwent magnetic resonance spectroscopy (MRS), which provides high sensitivity for identifying steatosis. Under this imaging protocol, MRS hepatic fat fraction (HFF) of >1.8% is equivalent to histological steatosis of >5%^(21)^.

Biopsied NAFLD cases were recruited as part of the European Paediatric NAFLD Registry (EU-PNAFLD, Clintrials.gov NCT:04190849)^(22)^, which was approved by the East Midlands - Nottingham 2 Research Ethics Committee (17/EM/0084). These children had been referred to specialist centres for paediatric hepatology (Birmingham Children’s Hospital (UK) and Bambino Gesù Children’s Hospital (Rome, Italy)) due to suspected advanced NAFLD and underwent liver biopsy for diagnosis of NAFLD and staging of disease.

As an exploratory analysis, we utilized data from the maximum number of available participants therefore no formal sample size calculation was performed.

### Plasma lipidomics analysis

For lipid profiling, plasma samples from all participants were analyzed by liquid chromatography with mass spectrometry detection (LC–MS) as described previously^(23)^ (and in Methods, Supplementary Digital Content 2).

Full details of statistical analysis, including comparison with adult NAFLD and annotation with GWAS loci is described in in Methods, Supplementary Digital Content 2. In brief, we tested the association between the plasma concentration of lipids and traits (e.g. alanine aminotransferase (ALT) levels, NAFLD Activity Score on biopsy) using linear regression. We then looked whether the number of double-bonds (saturation) or carbons (chain length) in each class of lipid (e.g. triglycerides) was associated with traits (i.e. meta-regression). Next, for the 72 lipids associated with histological severity of NAFLD, we looked up whether a similar association had been seen in adults with NAFLD. Lastly, we used other data from adults to see if these lipids are linked to cardiometabolic disease directly, or via genetic variants.

## Results

287 children were recruited to the study: 19 lean controls, 146 obese or overweight children, and 122 biopsied NAFLD cases (Table, Supplemental Digital Content 3). We studied a cohort of obese children and a group of children with NAFLD who had undergone biopsy in a specialist liver center. These three groups were brought together to understand differences in circulating lipid profile in severe NAFLD compared to children with obesity more typical of those seen in primary care.

The obese cohort included a spectrum from those with no evidence of metabolic dysfunction, through to those with marked insulin resistance (Figure, Supplemental Digital Content 4). Of the subset who had undergone liver MRS, 47% (45/90) had steatosis (i.e. >1.8% on MRS, which corresponds to >5% histological steatosis^(21)^).

In the biopsied NAFLD cases, a range of histological severity was observed (Table S1, Supplemental Digital Content 5). 100/122 (82%) of children had fibrosis and, though 5 children had stage 3 fibrosis, none were cirrhotic.

**Figure 1:**
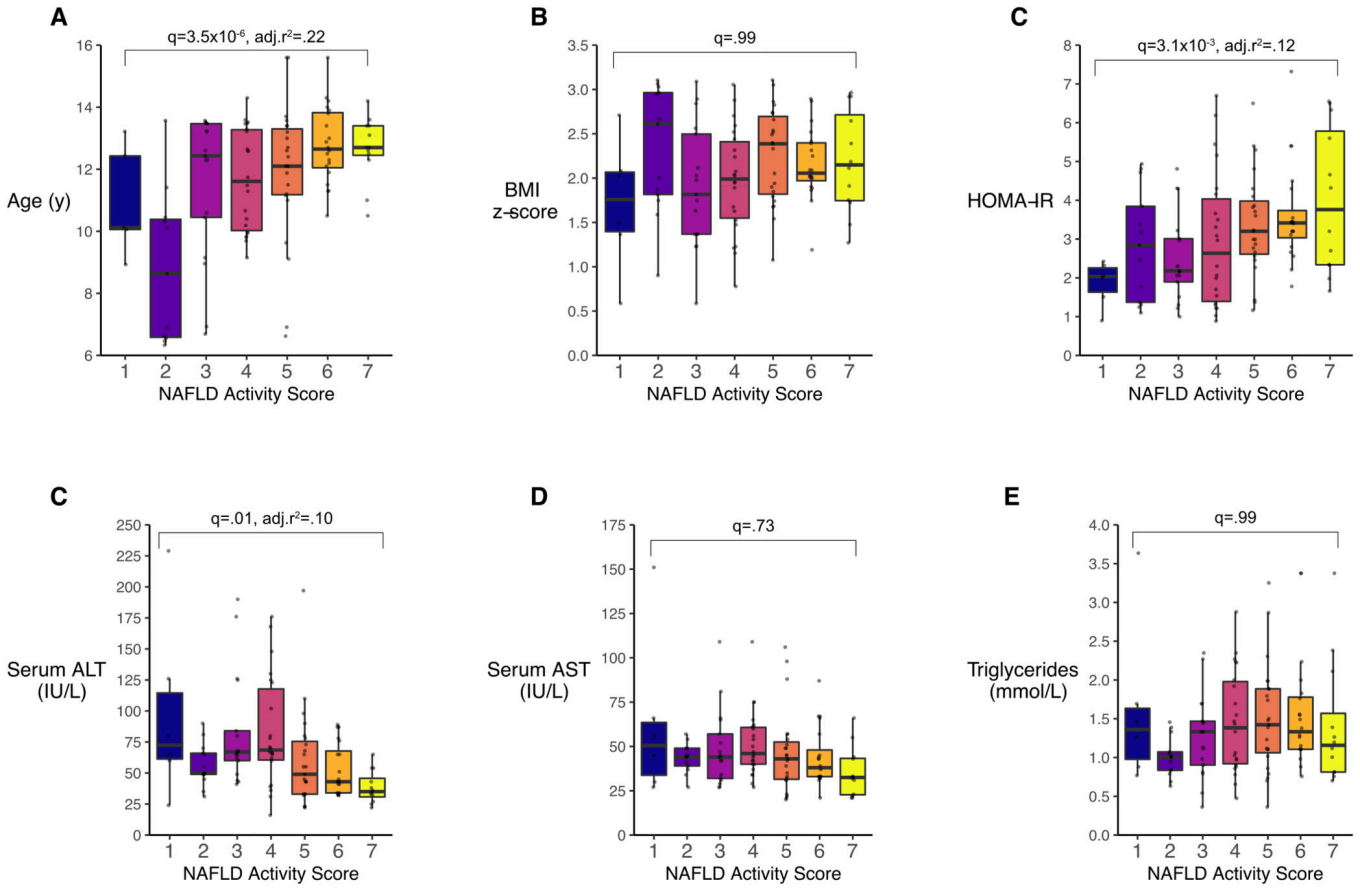
Associations between baseline characteristics and NAFLD Activity Score in children biopsied for NAFLD (n=122). Age (A) and homeostatic model of insulin resistance (HOMA-IR, C) were positively associated with NAFLD Activity Score, whilst serum alanine aminotransferase (C) was negatively associated. Body mass index (BMI, B) z-score, aspartate aminotransferase (D) and total serum triglycerides (E) were not associated with NAFLD Activity Score. Associations were tested using linear regression. q-values were derived using the Benjamini-Hochberg method where significance is q<.05.

Typical liver-related biochemistry (i.e. ALT, aspartate aminotransferase) and standard serum lipids (total triglycerides and cholesterol) were poor predictors of histological severity of NAFLD (Figure 1). Only age and insulin resistance were associated with histological severity of NAFLD.

**Figure 2:**
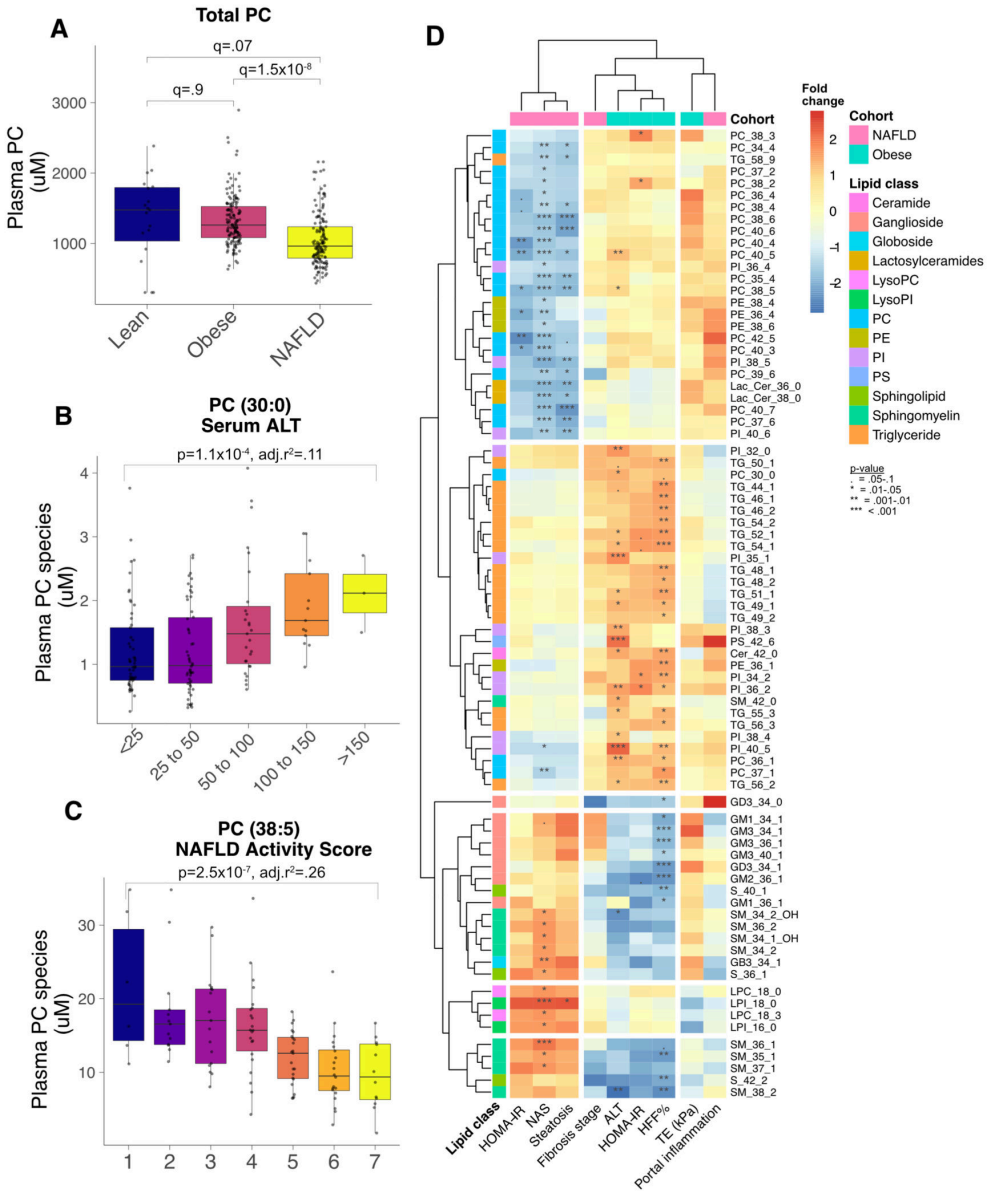
Associations between plasma lipid concentrations (μM) and fatty liver. (A) Total phosphatidylcholines (PC) by cohort. (B) Association between PC(30:0) and alanine aminotransferase (ALT) in obese cohort. (C) Association between PC(38:5) and NAFLD Activity Score. (D) Heatmap of all lipids (rows) associated with traits (columns) within either the obese (blue) or NAFLD (pink) groups using linear regression adjusted for age and sex. The cell color represents the beta regression coefficient for each analysis and stars illustrate p-values. Cer, ceramide; GB-, Globoside; GD-/GM-, Ganglioside; Lac-Cer, Lactosylceramide; LPC, lysophosphatidylcholine; (L)PI, (lyso-)phosphatidylinositol; SM, sphingomyelin; S-, sphingosine; TG, triglyceride.

Therefore, we performed plasma lipidomics to investigate whether lipids were associated with the severity of NAFLD. We tested each lipid against a range of metabolic and hepatic traits across both the obese cohort and biopsied NAFLD cases (Figure 2D). Similar patterns of lipid-trait associations were found for homeostatic model of insulin resistance (HOMA-IR), NAFLD Activity Score (NAS), and steatosis grade in the biopsied NAFLD cases. In the obese cohort, shared patterns of lipid-trait associations were found for HOMA-IR, hepatic fat fraction (HFF) based on MRS, and ALT. Baseline HOMA-IR was significantly correlated with these traits in both groups (Figure, Supplemental Digital Content 7D-E). i.e. HOMA-IR was positively correlated with NAS and hepatic fat fraction.

**Figure 3:**
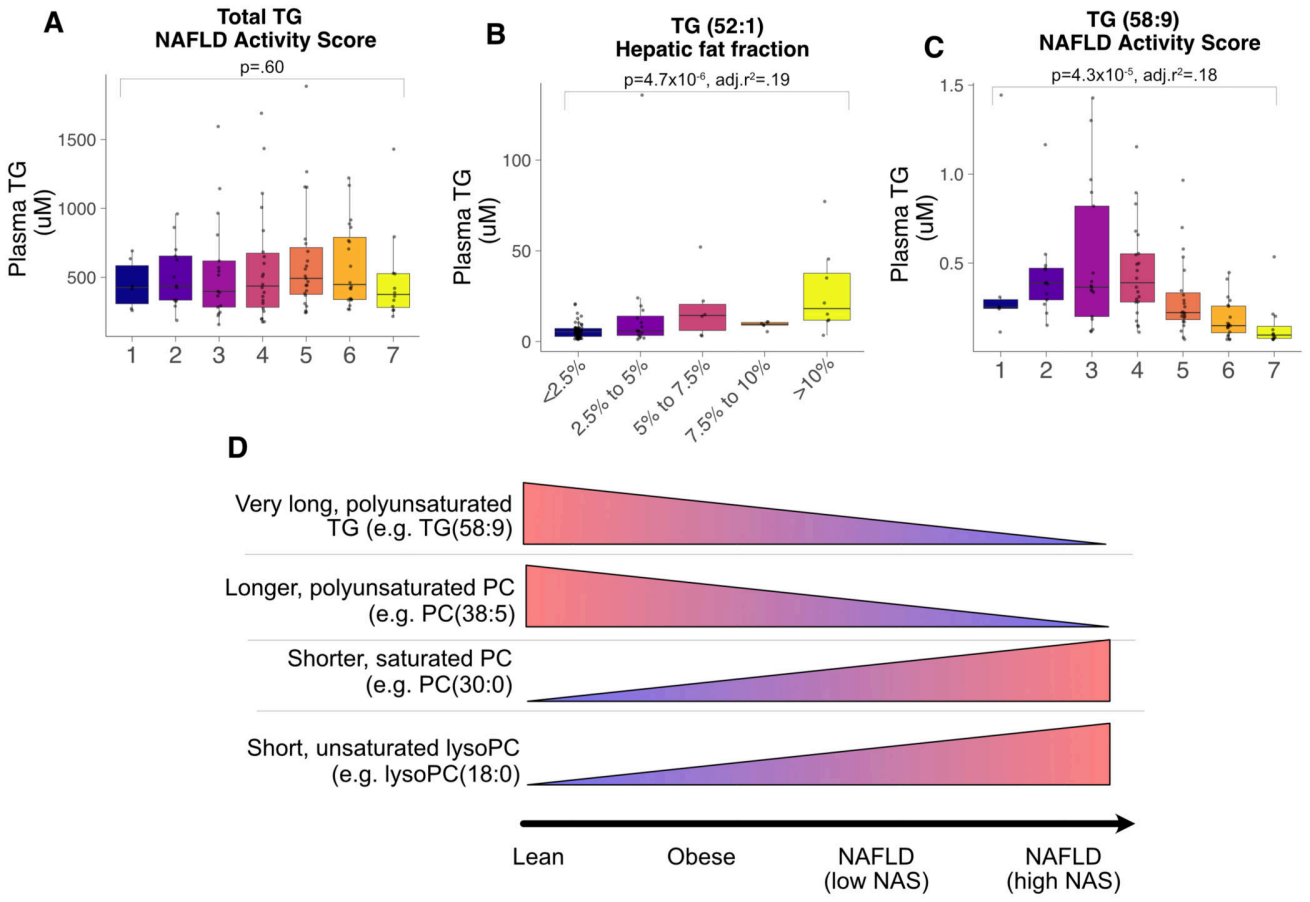
Associations between plasma triglyceride concentrations (μM) and fatty liver. (A) Total triglycerides (TG) by NAFLD Activity Score. (B) Association between TG(52:1) and hepatic fat fraction in obese cohort. (C) Association between TG(58:9) and NAFLD Activity Score. Associations were tested using linear regression adjusted for age and sex. (D) Summary of main lipid pathway perturbations.

Phosphatidylcholines (PC), a major component of lipid membranes, were lower in NAFLD cases than the obesity cohort (Figure 2A). However, not all PC species were lower. Shorter, saturated PC increased with higher ALT (Figure 2B). Whilst longer, polyunsaturated PC were inversely associated with NAS (Figure 2C) and HFF (Figure, Supplemental Digital Content 8). A similar pattern of carbon chain length and number of double bonds was seen for triglycerides (TG): higher liver fat and higher NAS were linked to lower very-long, polyunsaturated TG (Figure 3C & Figure, Supplemental Digital Content 9).

We also observed a positive association between levels of lysophosphatidylcholines (lysoPC) and severity of NAFLD, especially saturated lysoPC (e.g lysoPC(18:0)). LysoPC are formed from the hydrolysis of PC and can also function as signaling molecules. Total lysoPC increased from lean, to obese, to biopsied NAFLD cases (Figure, Supplemental Digital Content 10).

Overall, as severity of NAFLD increased, there were lower long, polyunsaturated TG and PC. This was coupled with higher short, saturated PC and lysoPC (Figure 3D).

Though these trends were consistent across the obesity cohort and biopsied NAFLD cases, there were a few notable differences. For example, sphingomyelin C36:1 (SM(36:1), a lipid involved in membranes and signaling) was positively associated with NAS but negatively associated with hepatic fat fraction in the obese cohort. Similar results were found for other sphingomyelin species (Figure 2D).

There was also a strong, negative association with polyunsaturated phosphatidylinositols (PI (e.g. PI(38:5), a signaling lipid) and NAFLD severity on biopsy (Figure, Supplemental Digital Content 10). However, there was no association found between PI and markers of NAFLD in the obesity cohort. Phosphatidylglycerols (PG) and total gangliosides, which were also lower in the NAFLD cases than the obesity cohort (Figure, Supplemental Digital Content 10).

In total, we identified 72 individual lipids associated with severity of NAFLD on biopsy (Table S5, Supplemental Digital Content 5). We found that 9 of the 72 lipids were associated with hepatic steatosis in a cohort of adults^(18)^. Many lipids were also associated with markers of the metabolic syndrome (e.g. higher body fat or HOMA-IR) in this adult cohort (Table S6, Supplemental Digital Content 5). The general patterns (i.e. lower polyunsaturated TG and PC in NAFLD) were replicated in the cohort of adults.

In order to understand the clinical relevance of these lipids we used data from a metabolite-wide association study^(19)^. We found that differences in these lipids associated with the development of cardiometabolic disease in adults (Table S6, Supplemental Digital Content 5).

Next, we identified GWAS-significant variants associated with these lipids using published data^(20)^. Many of these genetic variants were associated with (death from) cardiometabolic disease, higher fasting glucose, and body fat (Table 1). For example, lower plasma PC(36:4) was associated with: higher NAS in children; hepatic steatosis in adults (from Mann *et al*.^(18)^); all-cause mortality, diabetes, and cardiac failure in adults (from *Pietzner et al*.^(19)^); and, variants in *FADS1-2-3*, which are also independently linked to death from cardiovascular disease (Table 1).

## Discussion

There is a well-established association between paediatric NAFLD, insulin resistance, and obesity in childhood but the long-term metabolic outcomes of this condition have not yet been fully described. Moreover, due to differences in phenotype, it has been unclear whether children share the same perturbations of lipid metabolism as adults with NAFLD. We found that there are some similar patterns of altered lipids between adults and children and NAFLD (e.g. lower very-long chain polyunsaturated PC). However, most of the lipid signature of histological NAFLD in children (63 of 72 lipids) could not be replicated in a large cohort of adults.

Our analysis identified perturbation of multiple lipid groups, including PC and lysoPC. The majority of other lipidomic studies in NAFLD (done in adults^(11, 12, 24, 25)^) have also identified associations between PC species and NASH. Phosphatidylcholines are membrane-forming lipids and therefore in circulation their abundance is influenced by the concentration of lipoprotein particles. Hartley *et al*. observed lower concentrations of HDL in children with NAFLD, which could account for lower PC^(17)^. We also found higher levels of several saturated lysoPC to associated with NAFLD severity, similar to the findings by Puri *et al*.^(10)^. In addition, lysoPC(18:1) was one of the top species identified in a separate study that differentiated obese controls from children with NAFLD^(16)^. The lysoPC identified in the present study (lysoPC(16:0, 18:0)) could be generated by the activity of phospholipase A_2_ (PLA_2_) on PCs^(26, 27)^, therefore increased PLA2 activity might account for these observations. PLA2 is of particular interest in NAFLD (and cardio-metabolic disease) as its activity is thought to correlate with pro-inflammatory mediators, and presence of oxidized low density lipoprotein^(28)^. Whether (paediatric) NAFLD is an independent risk factor for atherosclerosis is a complex question^(29)^. We found several lipids altered in paediatric NAFLD to also be associated with cardiovascular disease, including via genetic variants, though the direction of causality remains unclear. Measuring other indirect markers of cardiovascular disease (blood pressure and carotid media intima thickness) in these patient groups, could provide further insight into the associations between CVD, lipid profile, and NAFLD.

There is a strong body of work implicating increased hepatic *de novo* lipogenesis (DNL) in NAFLD^(30)^ and insulin resistance^(31)^. We observed a strong correlation between liver fat and HOMA-IR. Lipidomic research has suggested that changes in triglycerides can serve as indirect evidence for altered rates of DNL^(32)^. Increased hepatic DNL is associated with a more short-chain, saturated TG and reduced long-chain, unsaturated TG^(33)^. These findings had been observed in a pilot study on obese teenagers^(34)^, which we have replicated. Overall, we consider our results to be reflective of hepatic insulin resistance but an alternative methodology, for instance using isotopically labeled substrates, would be needed to formally investigate DNL in paediatric NASH.

A wide range of associations have been identified in previous metabolite profiling studies in children with NAFLD. Several have found higher levels of (branched-chain) amino acids^(16, 17, 35–39)^. Levels of circulating amino acids, particularly branched chain amino acids, are correlated to (and causal of) insulin resistance^(40)^. Other studies have focused on gastro-intestinal tract-derived metabolites^(41)^, which also appear to show utility in differentiating controls from NAFLD, though are less effective in separating simple steatosis and NASH^(42)^. We did not attempt to use our data to derive a prediction algorithm due to the lack of a second, independent cohort of children biopsied for NAFLD.

The main strength of this study was the inclusion of participants from a spectrum of the metabolic syndrome. We also used histological severity of NAFLD as our main outcome, which is the gold standard of assessment and comparatively few liver biopsies are performed for fatty liver in children. Lastly, use of multiple publicly available datasets provided supporting clinical context and comparison with results from adults. This shows that the international lipidomics community is providing a strong evidence base for new studies to build on.

The principal limitation of this work is the lack of a second, biopsied cohort of children with NAFLD for validation of results. In addition, liver biopsy samples were not available for lipidomics, which would have improved our understanding of lipid metabolism at the level of the hepatocyte. However, studies that have included paired liver and plasma samples have found considerable overlap^(12)^. We also identified changes in the plasma consistent with previous reports from liver samples in adults^(10)^. Given the strong correlation between steatosis grade and NAS, and that comparatively few children in this cohort had severe fibrosis, these results are most informative of mild-moderate NAFLD driven by liver fat content. It should also be noted that our participants were primarily of non-Finnish European descent and therefore it is unclear to what extent these findings are generalisable to other ethnicities. Whilst we have illustrated several lipids of interest associated with the severity of paediatric NAFLD, further work, both practical and conceptual, would be required to validate these findings and progress this technique towards clinical utility. Lastly, as a cross-sectional study, we are unable to determine causality or define specific mechanisms for alterations of lipids. Future work that included longitudinal sampling could be used to investigate whether weight loss was associated with a normalization of lipid profile.

## Conclusion

Severity of paediatric NAFLD and insulin resistance are inversely associated with long-chain, polyunsaturated PC and TG, and positively associated with saturated lysoPC. These trends in saturation and chain length are linked cardiometabolic disease in adults. However, most individual lipid associations with paediatric NAFLD histology were not replicated in adults with steatosis.

## Supplementary Material

Supplemental Data File (doc, pdf, etc.)_1

Supplemental Data File (doc, pdf, etc.)_2

Supplemental Data File (doc, pdf, etc.)_3

Supplemental Data File (doc, pdf, etc.)_4

Supplemental Data File (doc, pdf, etc.)_5

Supplemental Data File (doc, pdf, etc.)_6

Supplemental Data File (doc, pdf, etc.)_7

Supplemental Data File (doc, pdf, etc.)_8

Supplemental Data File (doc, pdf, etc.)_9

Supplemental Data File (doc, pdf, etc.)_10

Supplemental digital content

## Figures and Tables

**Table 1:** Summary table of top lipids associated with histological severity of NAFLD with children. Lipids were included if: significantly associated with NAFLD Activity Score in children and showed a directionally consistent association with hepatic steatosis on ultrasound in an independent cohort of adults. Some lipids were significantly associated with disease outcomes in adults: (+) indicates a positive / (-) negative association between plasma lipid levels and trait. Five lipids have genome-wide significant loci and these variants are also independently associated with cardiometabolic traits on phenome-wide association studies. ALP, alkaline phosphatase; COPD, chronic obstructive pulmonary disease; CVD, cardiovascular disease; LysoPC, lyso-phosphatidylcholine; PC, phosphatidylcholine; TG, triglyceride.

Lipid	Association with NAS in children (current study)		Association with steatosis in adults (from Mann et al. 2020)		Associated disease outcomes in adults (from Pietzner et al. 2021)	GWAS-significant variants in or near genes	Cardiometabolic traits identified from PheWAS
	Beta	p-value	Beta	p-value			
LysoPC 16:0	0.20	1.1E-03	0.06	1.7E-05	Breast cancer (+), glaucoma (+), non-malignant skin cancer (+)	LIPC, MAF, MFSD2A	IHD, Death from PVD/mesenteric ischaemia/SAH, Metabolic syndrome
PC 36:4	-0.23	1.5E-04	-0.12	8.8E-03	Colon cancer (-), death (-), endometrial carcinoma (-), cardiac failure (-), diabetes mellitus (-), asthma (-), COPD (-), venous thromboembolism (-)	FADS1-2-3, FADS2	Death from CVD, Serum ALP, Arterial thrombosis, HbA1c, Fasting glucose, Metabolic syndrome, Colorectal cancer, Limb fat
PC 37:4	-0.19	9.4E-04	-0.21	1.2E-06		FADS2	Death from CVD, Serum ALP, Arterial thrombosis, HbA1c, Fasting glucose, Metabolic syndrome, Colorectal cancer, Limb fat
PC 38:3	-0.19	1.0E-03	-0.27	1.2E-09		FADS1-2-3	HbA1c, Fasting glucose, Metabolic syndrome, Colorectal cancer, Limb fat
PC 38:5	-0.29	2.5E-07	-0.22	1.4E-06	Death (-), asthma (-)	FADS2	Death from CVD, Serum ALP, Arterial thrombosis, HbA1c, Fasting glucose, Metabolic syndrome, Colorectal cancer, Limb fat
PC 38:6	-0.29	4.5E-07	-0.06	3.3E-06	Death (-), cardiac failure (-), peripheral arterial disease (-), asthma (-), COPD (-)		
PC 40:2	-0.19	2.0E-03	-0.31	6.2E-12			
PC 40:7	-0.29	5.8E-07	-0.23	3.8E-07	Breast cancer (-), death (-), diabete mellitus (-), asthma (-), COPD (-), lung cancer(-)		
TG 58:9	-0.24	4.3E-05	-0.09	4.2E-02			

## Data Availability

All data and results from analyses are contained within the manuscript and supplement. Code used in analyses is available from https://doi.org/10.5281/zenodo.4656980.

## What is Known

- Children with hepatic steatosis share features of the metabolic syndrome with adults who have the same condition
- Adults with fatty liver are at increased risk of diabetes and cardiovascular disease
- Changes in the plasma lipid profile of adults associates with risk of cardiometabolic diseases

## What is New

- Changes in plasma triglycerides and phosphatidylcholines are associated with severity of fatty liver disease and insulin resistance in children
- Similar patterns of lipids are associated with cardiometabolic disease in adults
- Many of the specific lipids associated with paediatric NAFLD are unique to children

## Abbreviations

ALP: alkaline phosphatase
ALT: alanine aminotransferase
AST: aspartate aminotransferase
BMI: body mass index
Cer: ceramide
COPD: chronic obstructive pulmonary disease
CVD: cardiovascular disease
DNL: *de novo* lipogenesis
GB-: Globoside
GD-/GM-: Ganglioside
GWAS: genome-wide association study
HFF: hepatic fat fraction
HOMA-IR: homeostatic model of insulin resistance
Lac-Cer: lactosylceramide
LC-MS: liquid chromatography mass spectrometry
(lyso-)PC: (lyso-)phosphatidylcholine
(L)PI: (lyso-)phosphatidylinositol
MRS: magnetic resonance spectroscopy
mWAS: metabolite-wide association study
NAFLD: non-alcoholic fatty liver disease
NASH: non-alcoholic steatohepatitis
NAS: NAFLD Activity Score
PC: phosphatidylcholine
PheWAS: phenome-wide association study
PI: phosphatidylinositol
PLA2: phospholipase A2
S-: sphingosine
SM: sphingomyelin
TG: triglyceride
