# Supplementary figures and images for: Comparison of the lipidomic signature of fatty liver in children and adults: a cross-sectional study
Source: J Pediatr Gastroenterol Nutr. Author manuscript; Available in PMC 2022 Jul 8. (PMC7613028; doi:10.1097/MPG.0000000000003418)

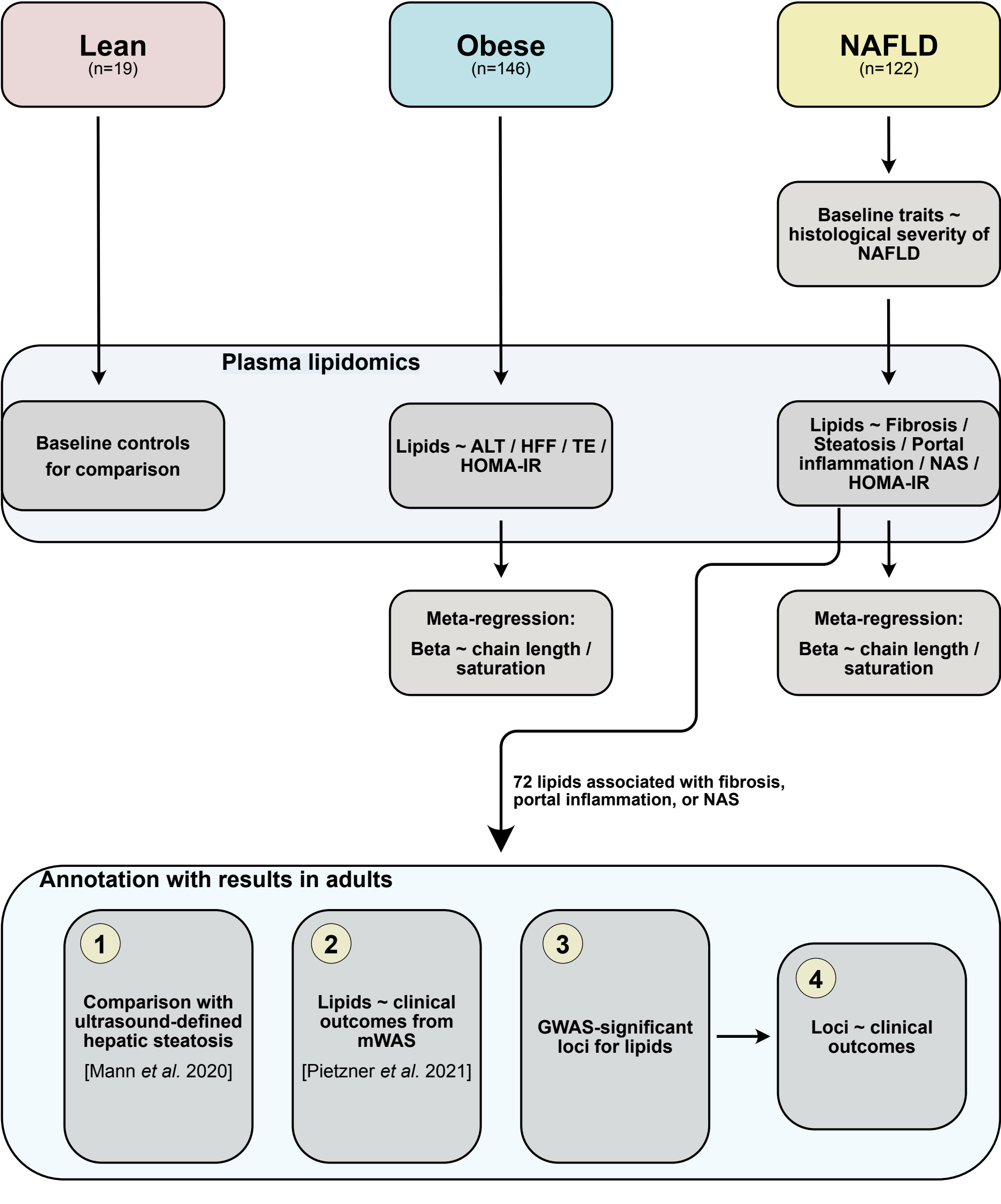

Supplement: Supplemental Data File (doc, pdf, etc.)_1 [file EMS143665-supplement-Supplemental_Data_File__doc__pdf__etc___1.pdf]

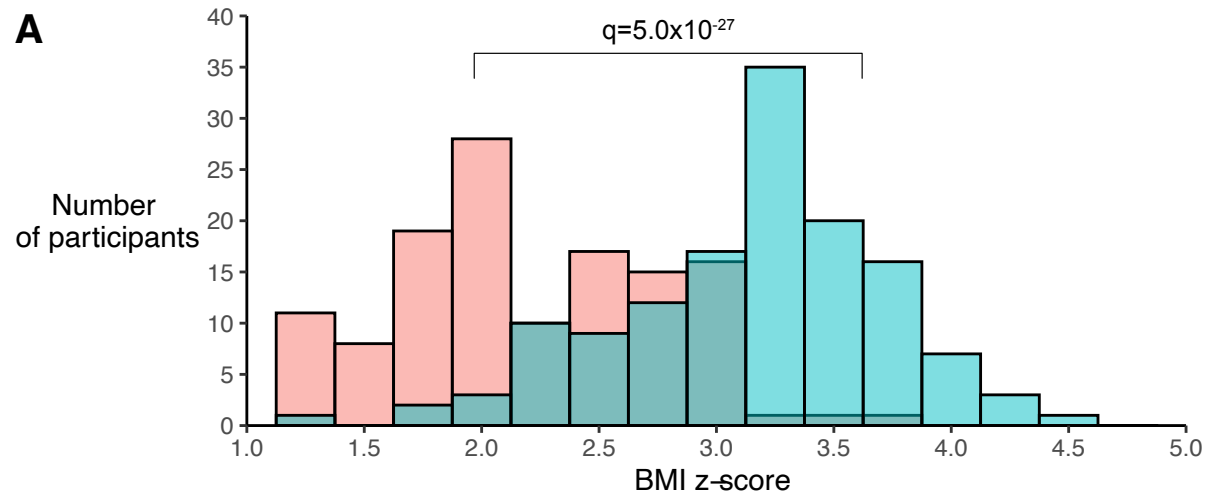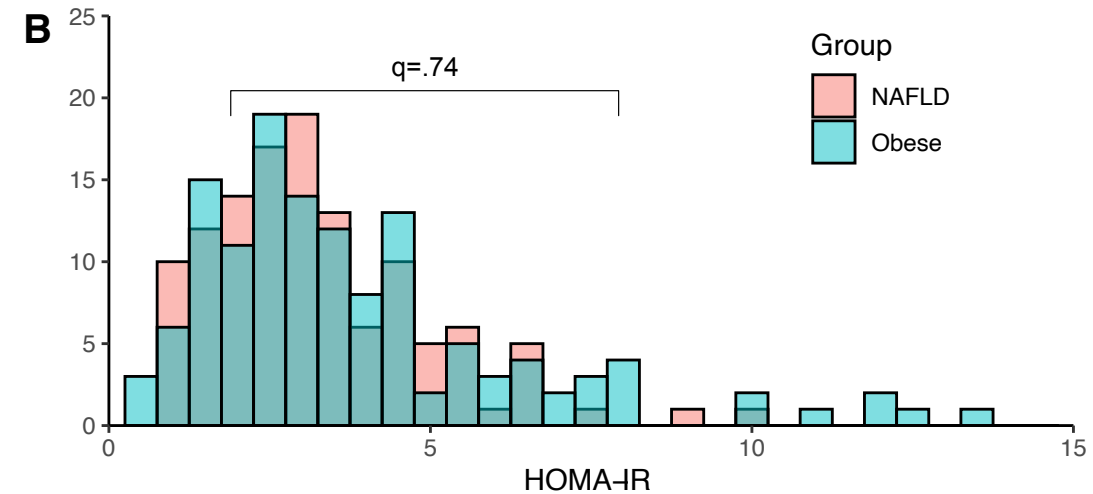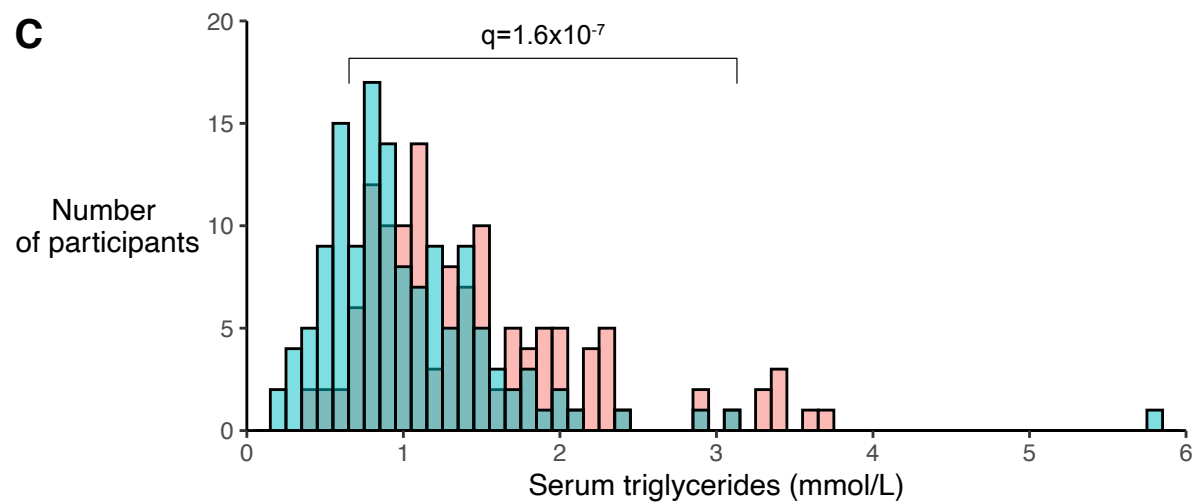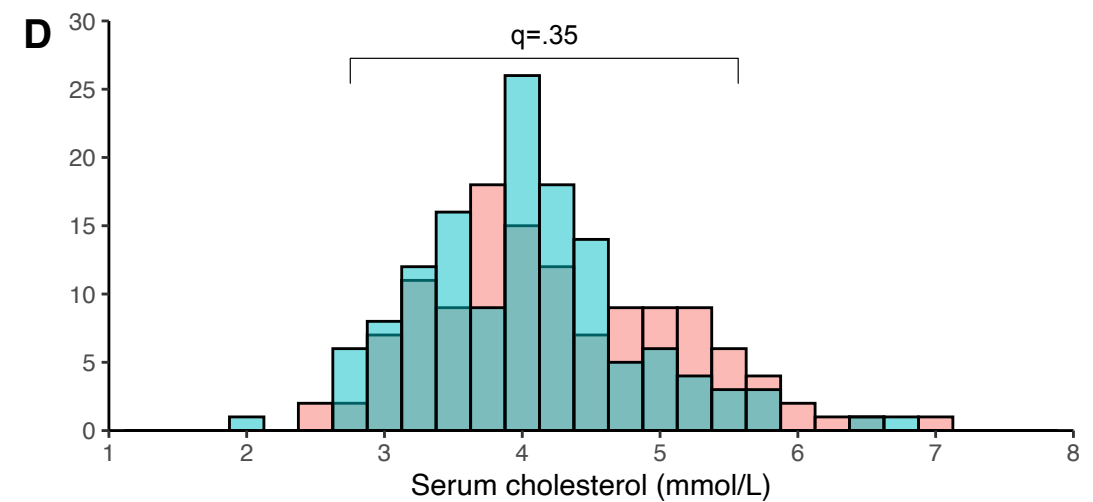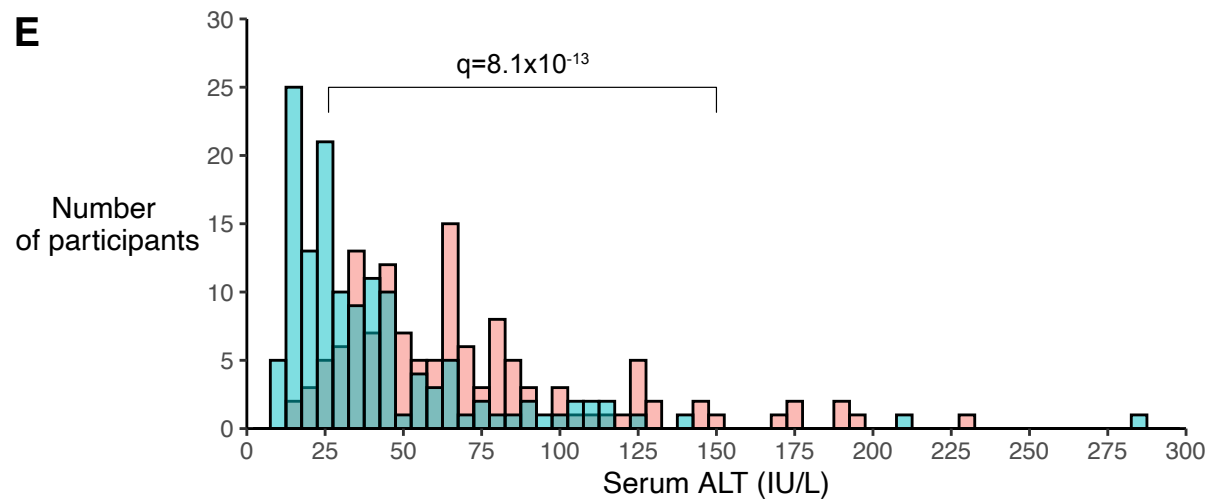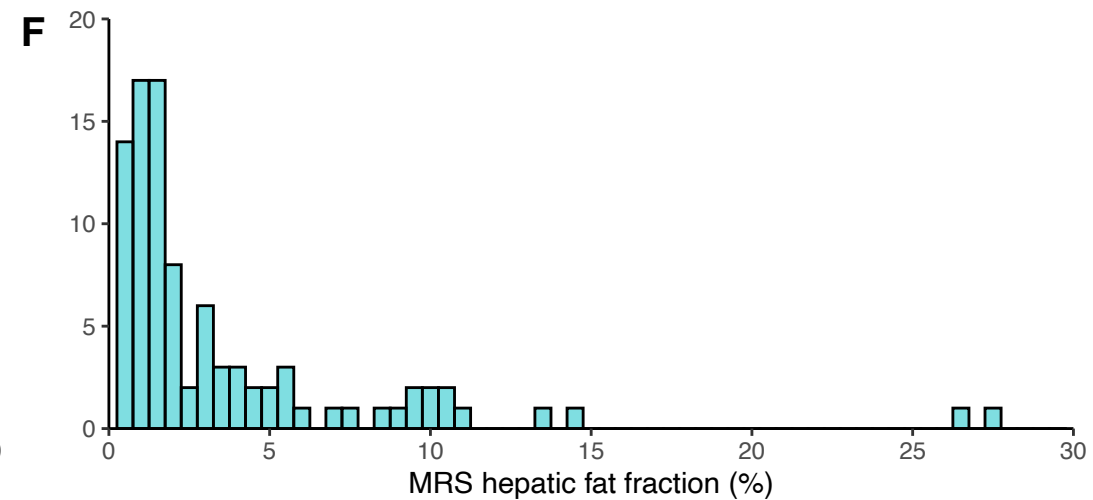

Supplement: Supplemental Data File (doc, pdf, etc.)_3 [file EMS143665-supplement-Supplemental_Data_File__doc__pdf__etc___3.pdf]

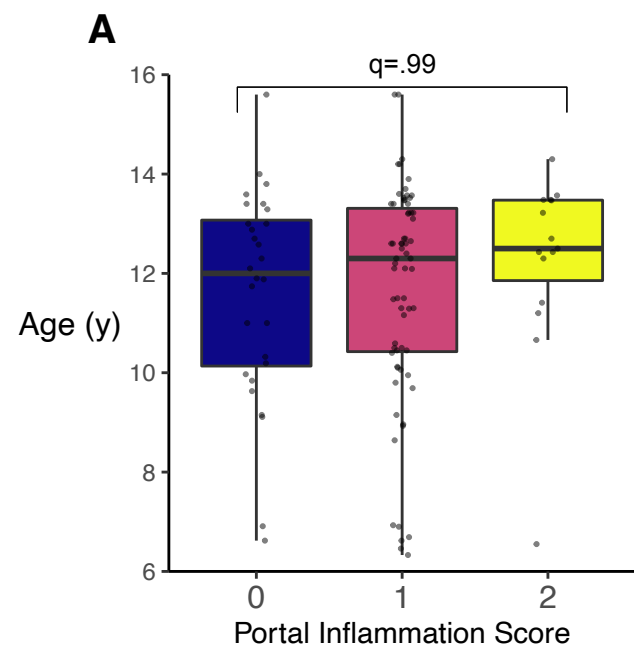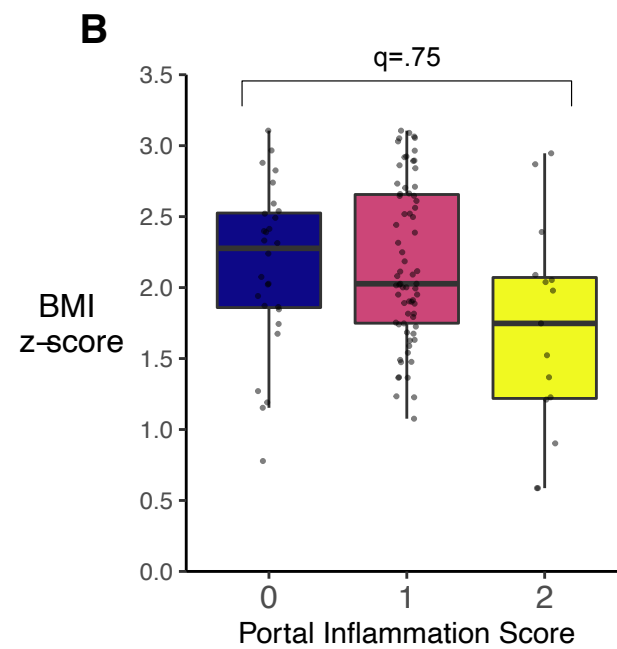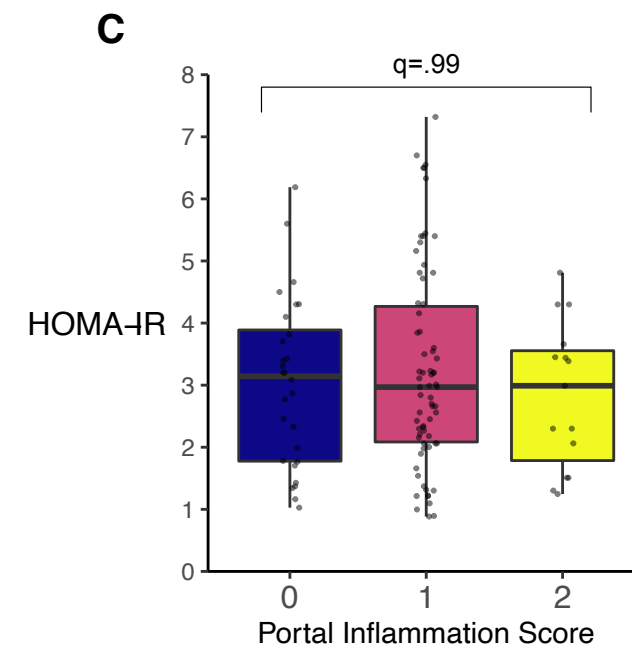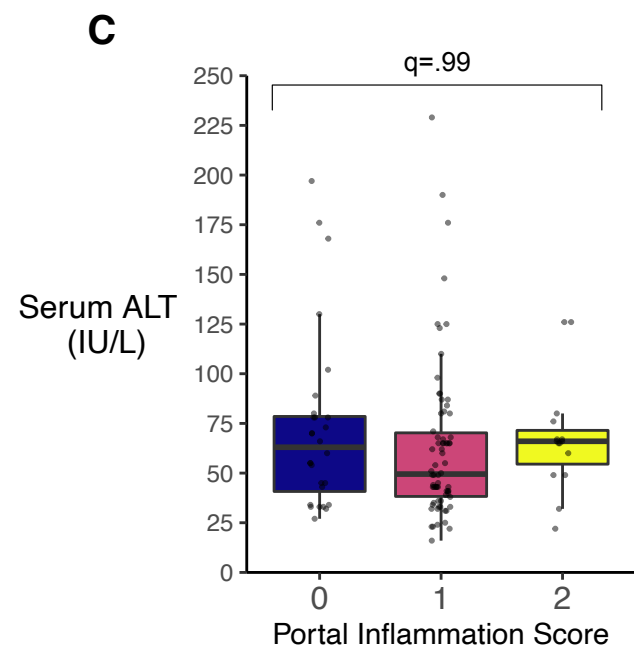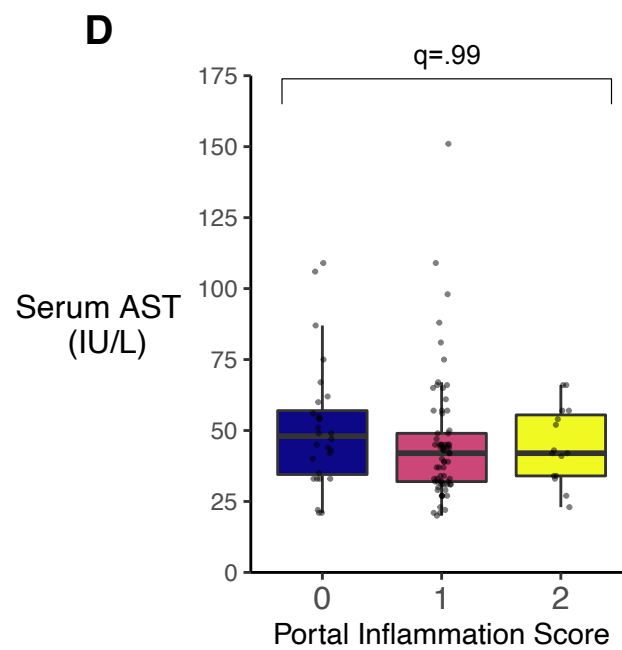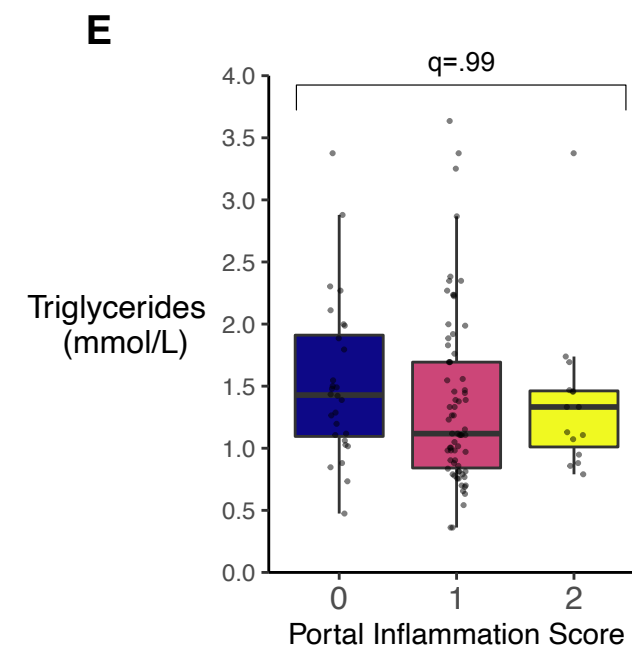

Supplement: Supplemental Data File (doc, pdf, etc.)_4 [file EMS143665-supplement-Supplemental_Data_File__doc__pdf__etc___4.pdf]

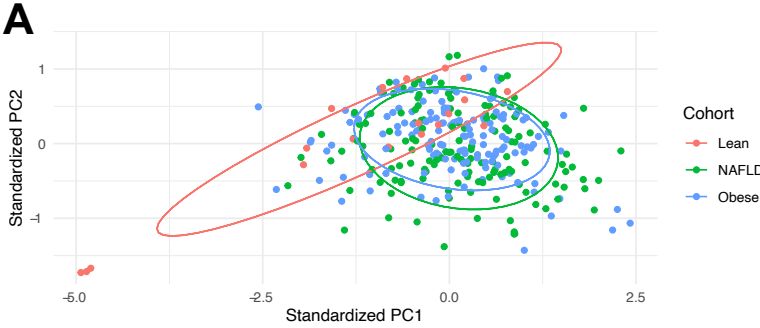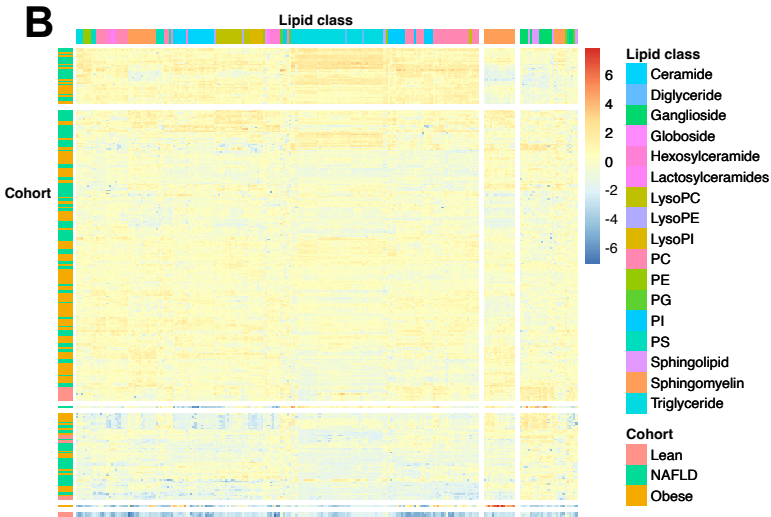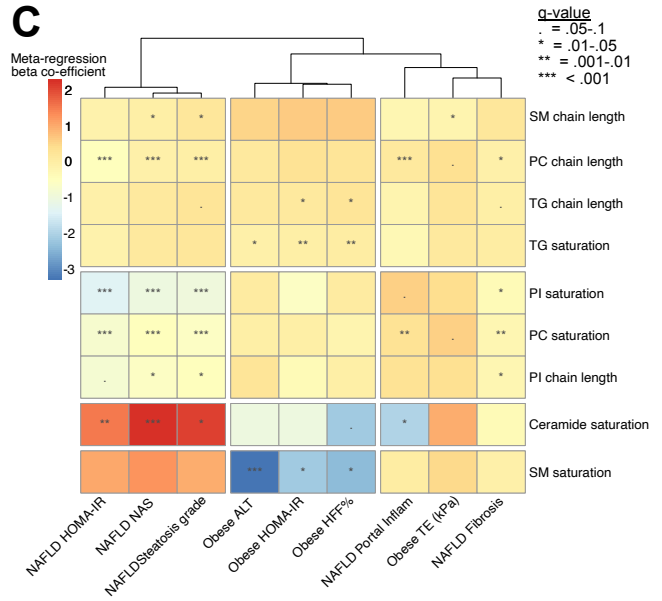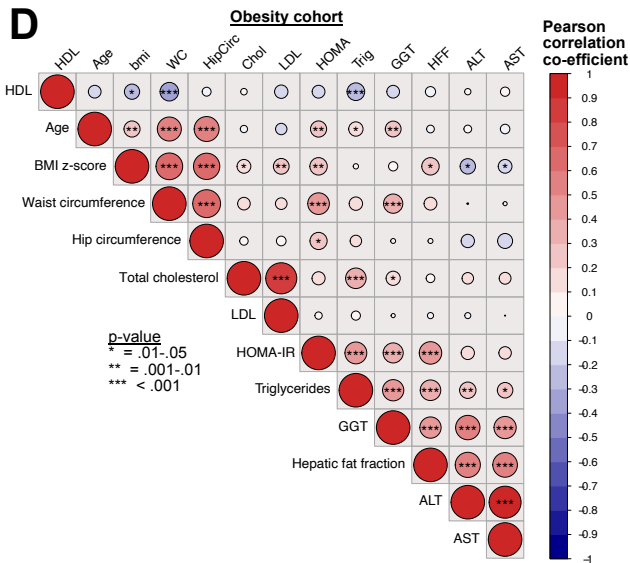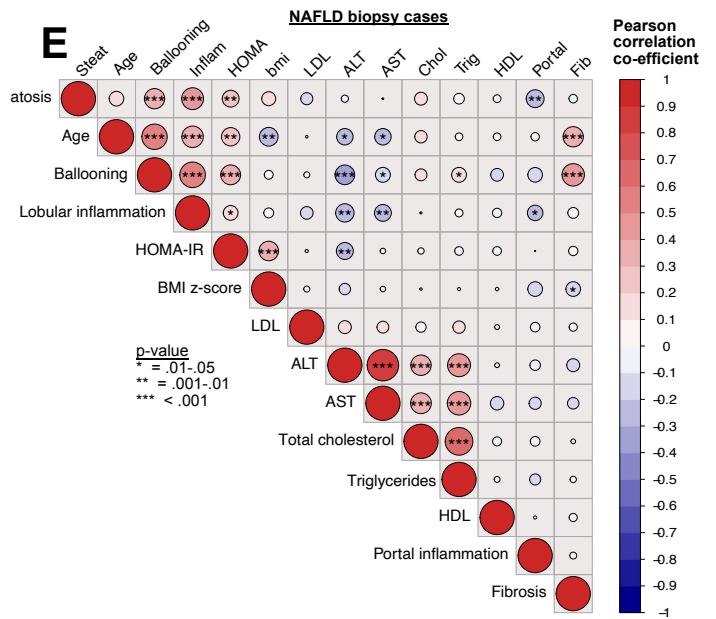

Supplement: Supplemental Data File (doc, pdf, etc.)_7 [file EMS143665-supplement-Supplemental_Data_File__doc__pdf__etc___7.pdf]

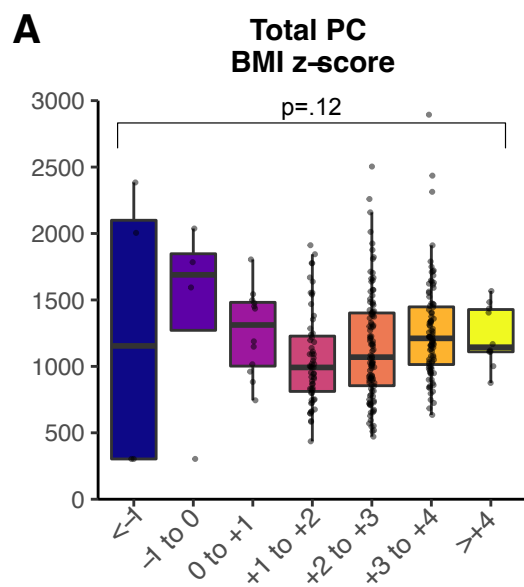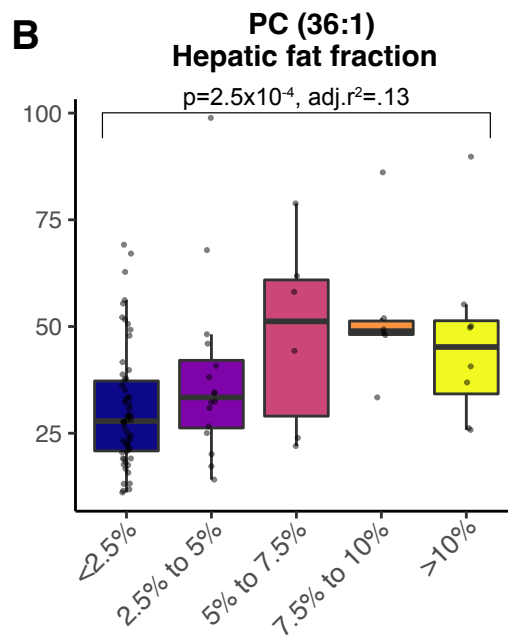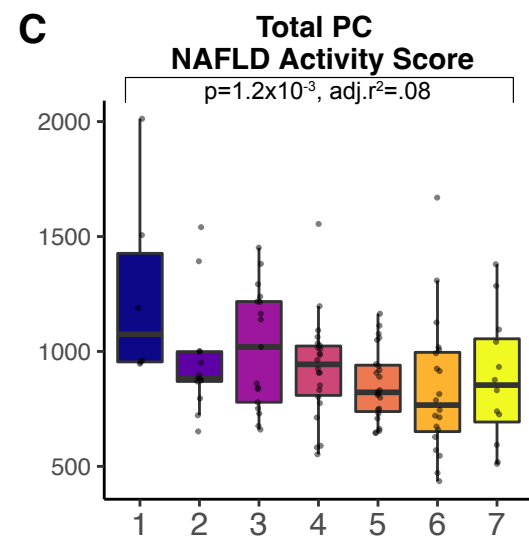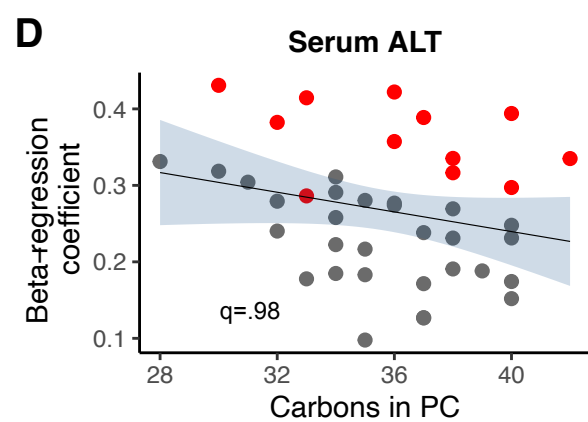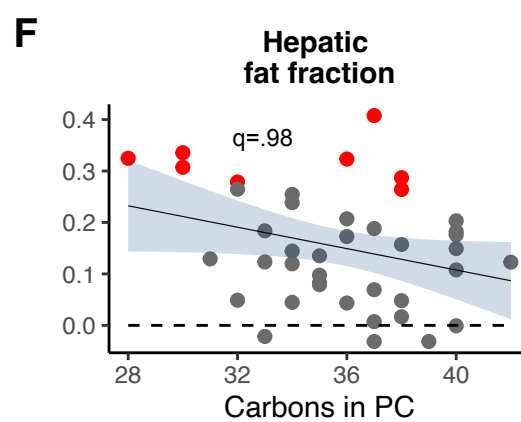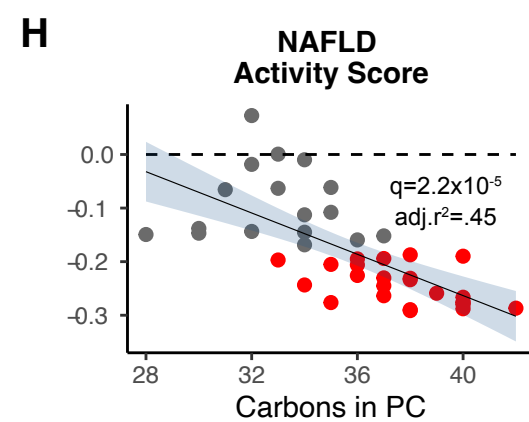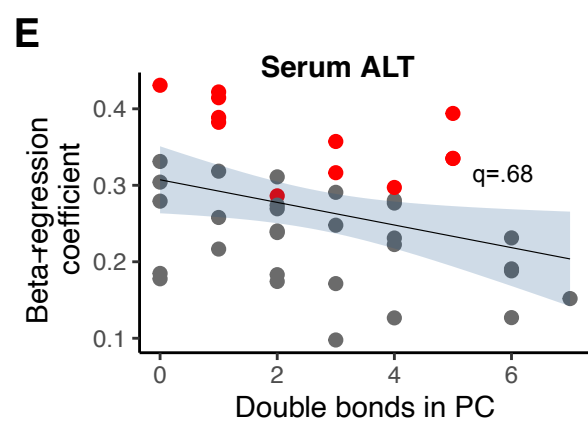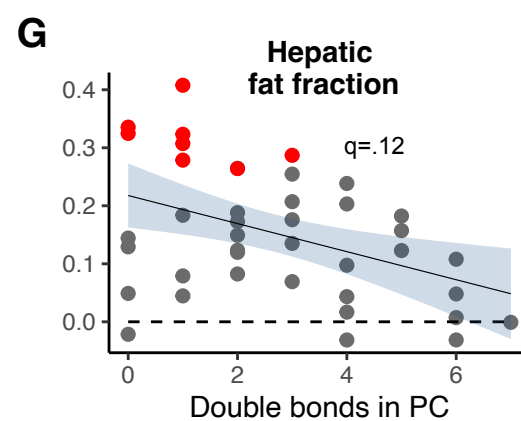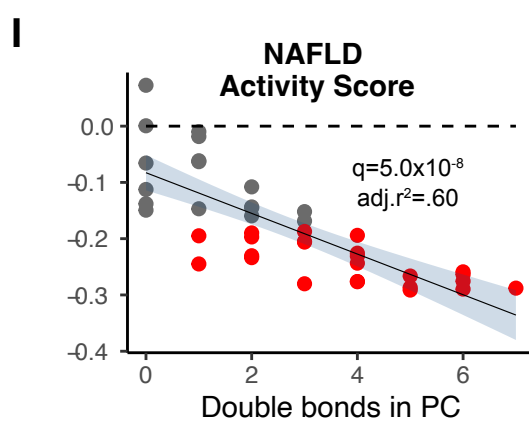

Supplement: Supplemental Data File (doc, pdf, etc.)_8 [file EMS143665-supplement-Supplemental_Data_File__doc__pdf__etc___8.pdf]

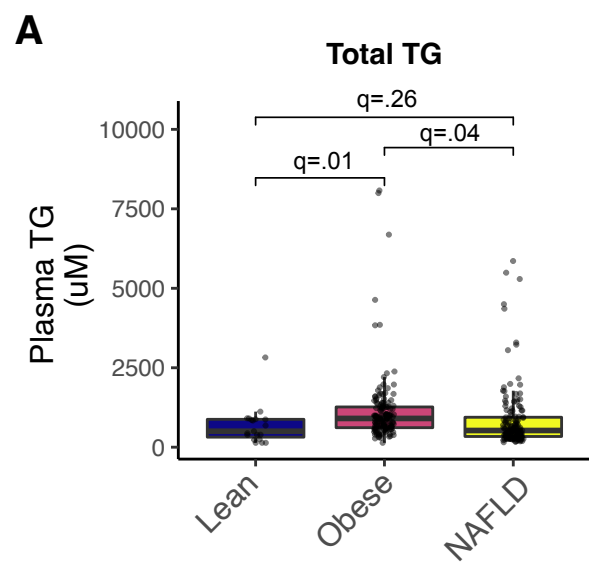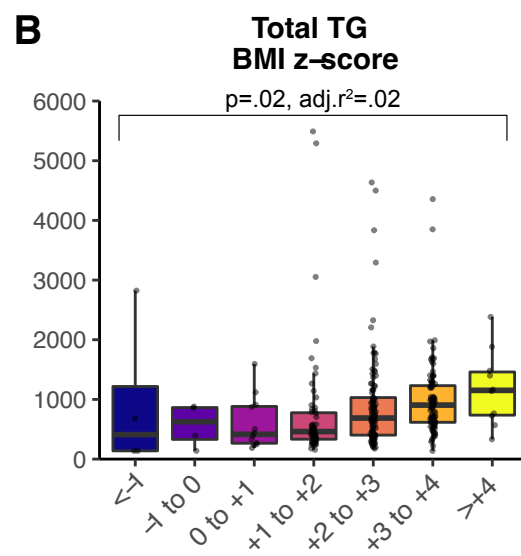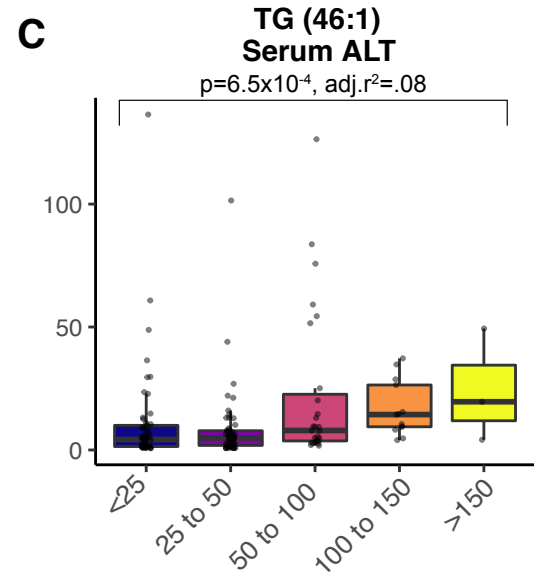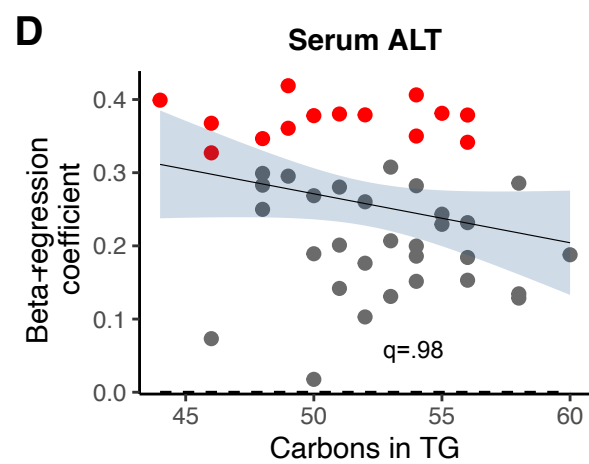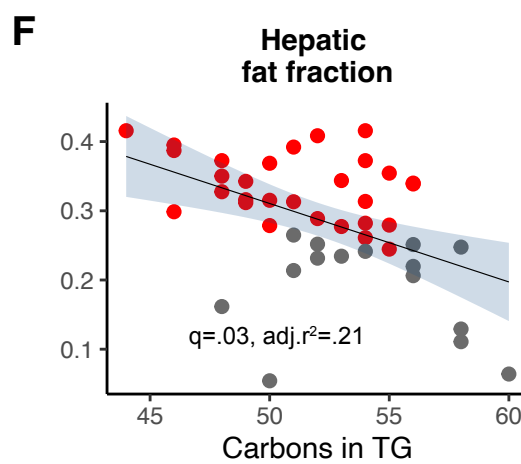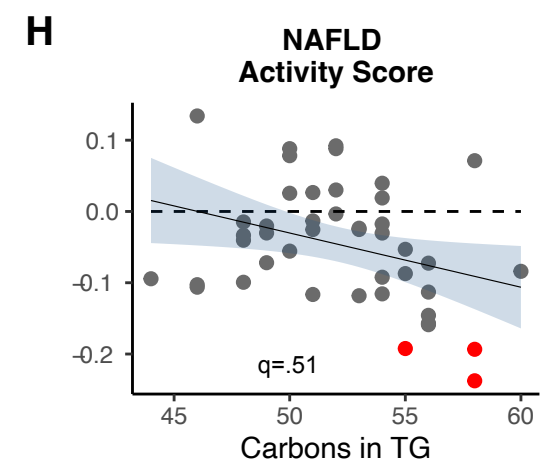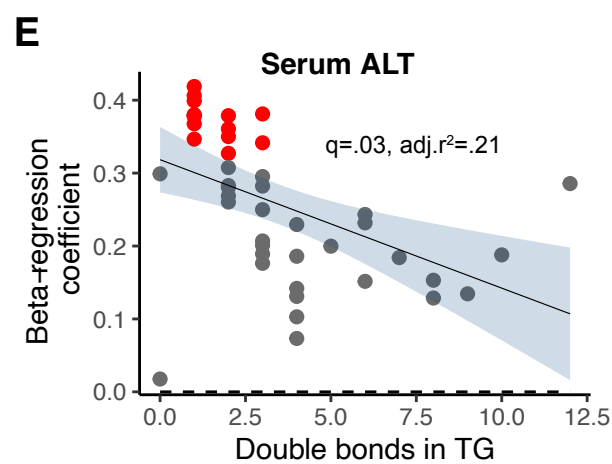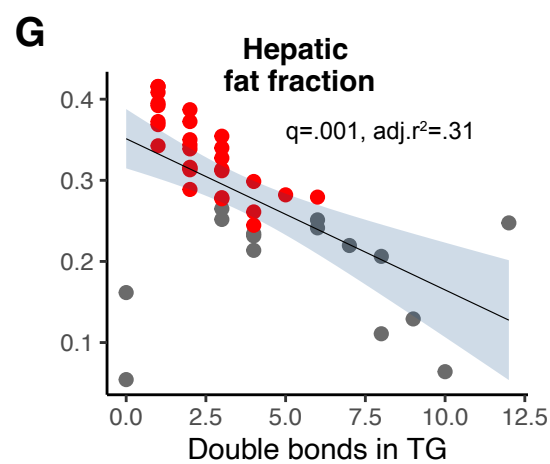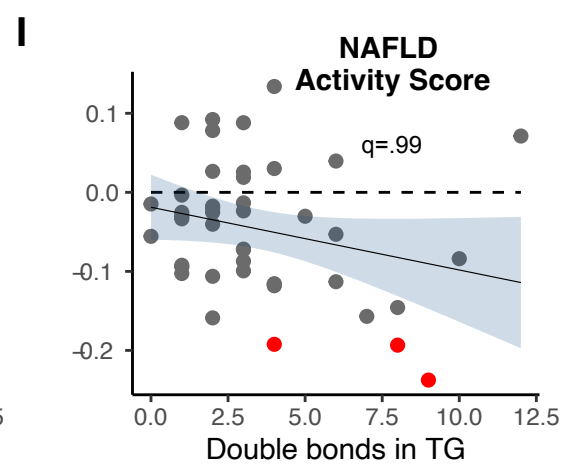

Supplement: Supplemental Data File (doc, pdf, etc.)_9 [file EMS143665-supplement-Supplemental_Data_File__doc__pdf__etc___9.pdf]

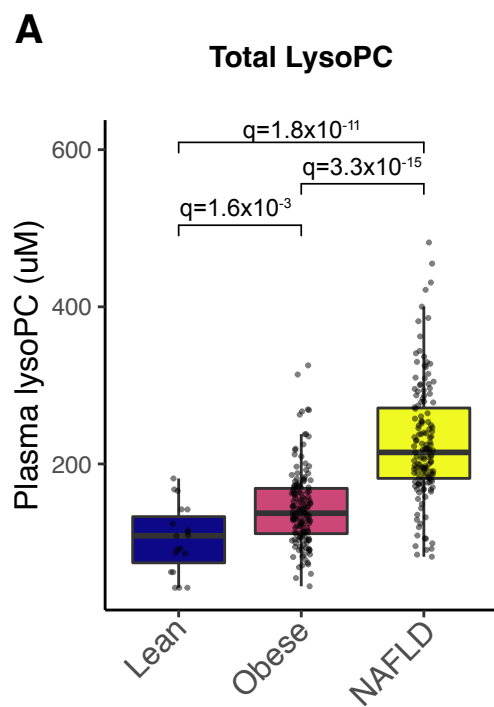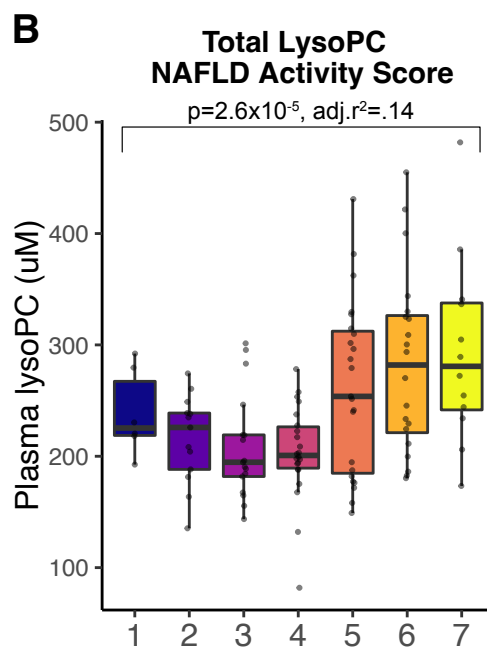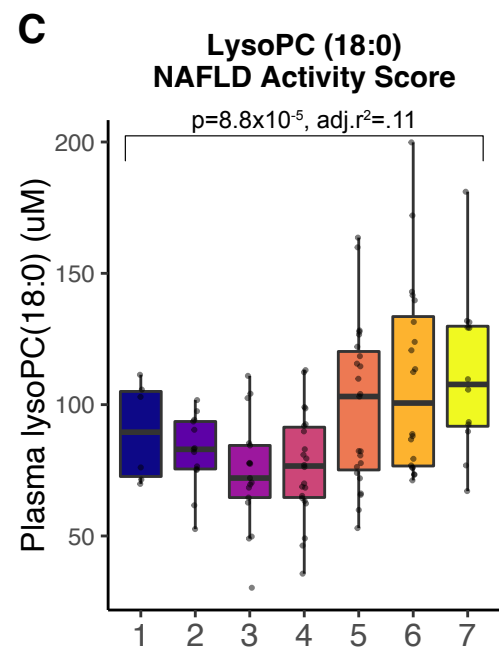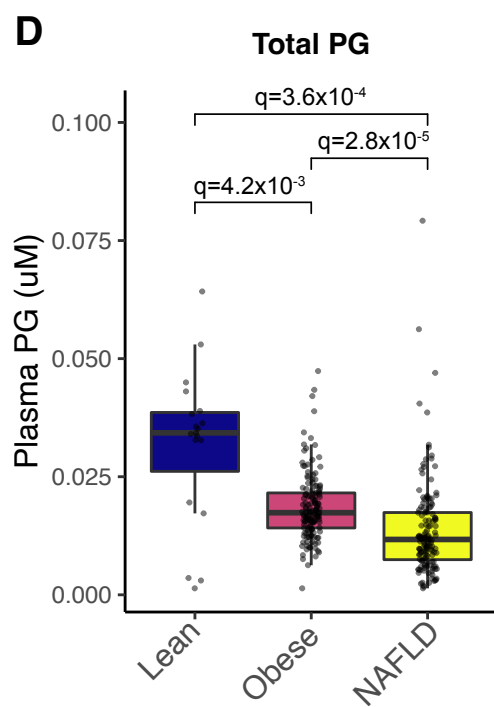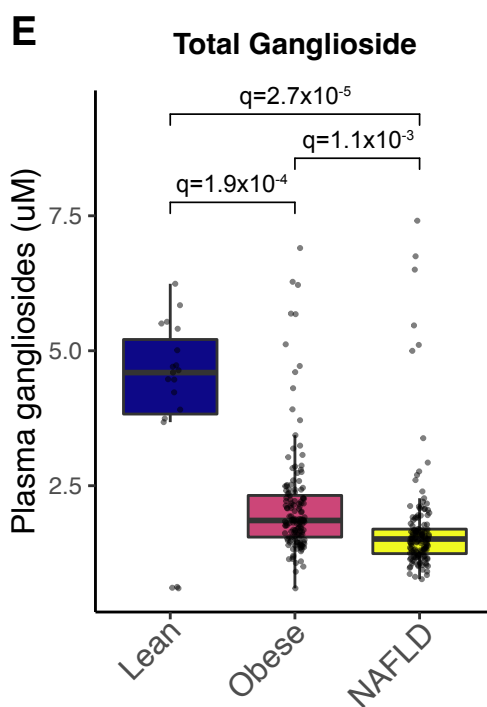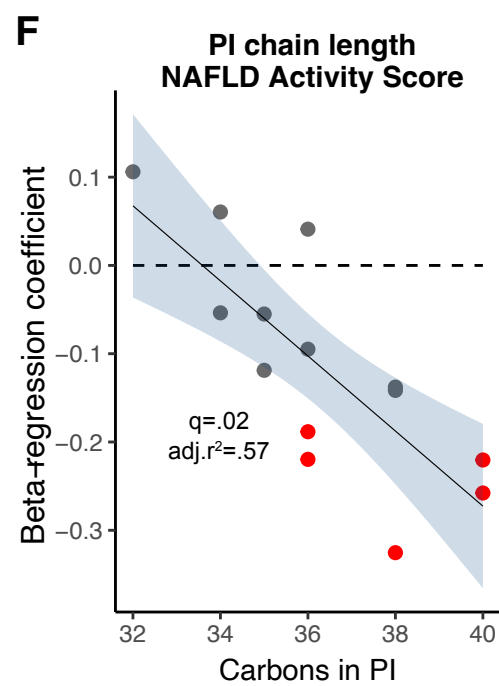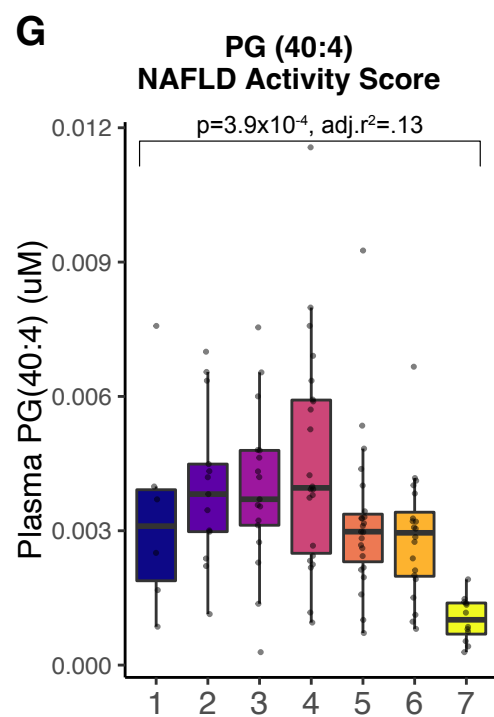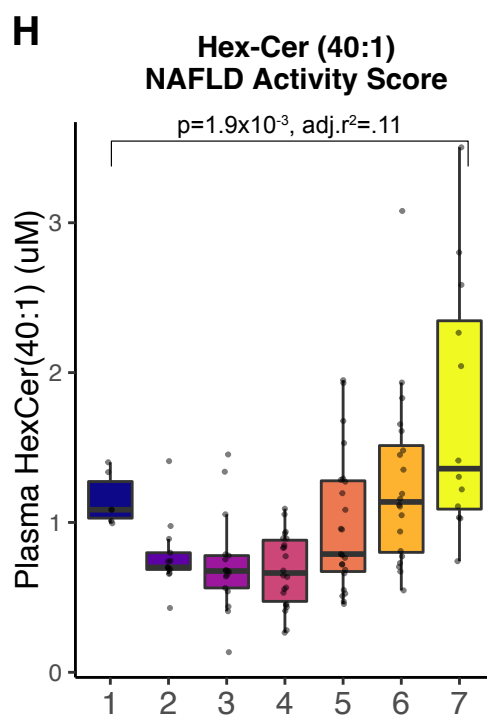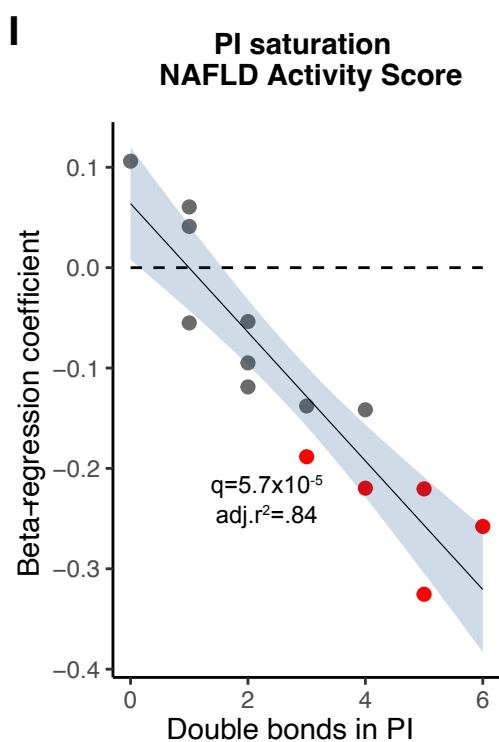

Supplement: Supplemental Data File (doc, pdf, etc.)_10 [file EMS143665-supplement-Supplemental_Data_File__doc__pdf__etc___10.pdf]
