# Supplementary material for: Comparison of the lipidomic signature of fatty liver in children and adults: a cross-sectional study
Source: J Pediatr Gastroenterol Nutr. Author manuscript; Available in PMC 2022 Jul 8. (PMC7613028; doi:10.1097/MPG.0000000000003418)
Supplement: Supplemental Data File (doc, pdf, etc.)_2 [file EMS143665-supplement-Supplemental_Data_File__doc__pdf__etc___2.docx]

|  | **Overall** | **Lean controls** | **Obese cohort** | **NAFLD cases** | **q-value** |
| --- | --- | --- | --- | --- | --- |
| n | 287 | 19 | 146 | 122 |  |
| Age (years) | 13.0 (2.8) | 11.0 (3.4) | 14.2 (2.4) | 12.0 (2.5) | 1.00E-10 |
| Female sex | 162 (52.4) | 10 (52.6) | 76 (52.1) | 76 (52.8) |  |
| BMI z-score | 2.48 (1.1) | -0.04 (1.2) | 3.16 (0.6) | 2.15 (0.6) | 5.00E-27 |
| HOMA-IR | 3.6 (2.2) |  | 3.8 (2.6) | 3.2 (1.7) | 0.74 |
| Hypertension, n/total (%) | 74/233 (32) |  | 59/119 (50) | 15/114 (13) | 2.40E-9 |
| Triglycerides (mmol/L) | 1.2 (0.7) |  | 1.0 (0.6) | 1.4 (0.7) | 1.60E-07 |
| Total cholesterol (mmol/L) | 4.2 (0.8) |  | 4.0 (0.8) | 4.3 (0.9) | 0.35 |
| HDL cholesterol (mmol/L) | 1.1 (0.3) |  | 1.1 (0.3) | 1.1 (0.3) | 1 |
| LDL cholesterol (mmol/L) | 2.5 (0.6) |  | 2.5 (0.7) | 2.6 (0.5) | 0.07 |
| ALT (IU/L) | 55.3 (41.8) |  | 41.9 (37.4) | 69.3 (41.7) | 8.1E-13 |
| AST (IU/L) | 38.6 (20.7) |  | 30.0 (14.5) | 47.7 (22.3) | 2.3E-16 |
| GGT (IU/L) | 25.4 (12.8) |  | 24.3 (11.8) | 36.4 (17.3) | 9.0E-03 |
| ALP (IU/L) | 200.8 (122.3) |  | 184.3 (113.6) | 260.1 (139.7) | 0.66 |
| Hepatic fat fraction on MRS (%) | 3.6 (4.7) |  | 3.6 (4.7) |  |  |

**Table 1. Baseline characteristics of participants included in the study.** Lean children (n=19) were those undergoing endoscopy who had no evidence of gastrointestinal pathology. Obesity cohort (n=146) comprised children who were overweight or obese referred and for clinical assessment. The NAFLD cases (n=122) were children with suspected severe paediatric NAFLD who underwent liver biopsy. q-values represent false-discovery rate correct p-values between obesity and NAFLD cohorts, using unpaired t-tests for continuous traits and chi-squared for sex. Data represents mean (standard deviation) for continuous traits and number (%) for categorical traits. ALP, alkaline phosphatase; ALT, alanine aminotransferase; AST, aspartate aminotransferase; BMI, body mass index; GGT, gamma-glutamyl transferase; HDL, high density lipoprotein; HOMA-IR, homeostatic model of insulin resistance; LDL, low density lipoprotein; MRS, magnetic resonance spectroscopy.
