# Supplementary material for: Comparison of the lipidomic signature of fatty liver in children and adults: a cross-sectional study
Source: J Pediatr Gastroenterol Nutr. Author manuscript; Available in PMC 2022 Jul 8. (PMC7613028; doi:10.1097/MPG.0000000000003418)
Supplement: Supplemental Data File (doc, pdf, etc.)_5 [file EMS143665-supplement-Supplemental_Data_File__doc__pdf__etc___5.docx]

**Supplementary Methods**

## Inclusion and exclusion criteria

All participants were 5-18 years old at the time of inclusion.

Lean controls: BMI z-score <1.04, normal serum liver biochemistry, and no clinical evidence of liver disease. The obesity cohort all had a BMI z-score >1.64 (except for one participant who was overweight with BMI z-score 1.2) and no evidence of secondary causes of obesity (e.g. hypothyroidism). Biopsied NAFLD cases: NAFLD diagnosed by liver histology, plus exclusion of secondary causes using blood tests: normal range ceruloplasmin, ferritin, alpha-1-antitrypsin; normal immunoglobulins, negative antinuclear antibody, and negative anti-smooth muscle antibody; and negative screening for chronic viral hepatitis.

No participant was treated with drugs that directly alter lipid metabolism (e.g. statins).

Prior to inclusion, participants had a clinical and biochemical assessment to look for genetic causes of dyslipidaemia or severe insulin resistance. However, sequencing of genes implicated in familial dyslipidaemia (e.g. *LDLR*, *PCSK9*) was not performed, therefore we cannot rule out inclusion of some individuals with all known variants in such genes.

## Clinical and laboratory investigations

Lean controls had height and weight measured and fasting blood obtained but no further samples or investigations were performed for the purpose of this study.

The obesity cohort and biopsied NAFLD cases both underwent baseline characterization with clinical assessment and anthropometric measurements including waist/hip circumferences and blood pressure. Fasting blood was obtained and plasma was frozen at -80 degree Celsius for use in all analyses. The homeostatic model assessment of insulin resistance (HOMA-IR) was derived using fasting insulin (µU/L) x fasting glucose (nmol/L) / 22.5. A subset of the obesity cohort (n=95) underwent magnetic resonance spectroscopy (MRS) for quantification of hepatic fat and a separate subset (n=42) had transient elastography (TE) performed to non-invasively test for signs of NAFLD. Steatosis was defined as MRS >1.8%, which corresponds with >5% fat containing hepatocytes on liver histology^(1)^.

## Liver biopsies

All NAFLD cases underwent liver biopsy whereas liver biopsy was not performed in control participants or the obesity cohort. Liver biopsy was performed in accordance with ESPGHAN guidelines^(2)^. Biopsies were obtained percutaneously and considered adequate if there was minimal fragmentation and at least 20 mm long. Liver biopsy was scored according to NASH CRN criteria^(3, 4)^, including assessment of portal inflammation as none (0), mild (1), or more than mild (2), and calculation of the NAFLD Activity Score (NAS). Samples from liver biopsy were used for histological assessment only; no tissue was available for lipidomic (or transcriptomic) analysis.

## Plasma lipidomics analysis

All solvents and additives were of HPLC grade or higher and purchased from Sigma Aldrich (Haverhill, Suffolk, UK) unless otherwise stated.

The protein‑precipitation liquid extraction protocol has been described previously^(5)^. Briefly, 50 µL of plasma was transferred into a 2 mL screw cap Eppendorf plastic tube (Eppendorf, Stevenage, UK). Immediately, 650 µL of chloroform was added to each sample, followed by thorough mixing. Then, 100 µL of the LIPID-IS (5 µM in methanol), 100 µL of the CARNITINE-IS (5 µM in methanol) and 150 µL of methanol was added to each sample, followed by thorough mixing. Then, 400 µL of acetone was added to each sample. The samples were vortexed and centrifuged for 10 minutes at ~20,000 g to pellet any insoluble material. The supernatant was pipetted into separate 2 mL screw cap amber-glass auto-sampler vials (Agilent Technologies, Cheadle, United Kingdom). The organic extracts were dried down to dryness using a Concentrator Plus system (Eppendorf, Stevenage, UK) run for 60 minutes at 60 degree Celsius. The samples were reconstituted in 100 µL of 2: 1: 1 (propan‑2‑ol, acetonitrile and water, respectively) then thoroughly vortex. The reconstituted sample was transferred into a 250 μL low-volume vial insert inside a 2 mL amber glass auto-sample vial ready for liquid chromatography with mass spectrometry detection (LC-MS) analysis.

Full chromatographic separation of intact lipids was achieved using Shimadzu HPLC System (Shimadzu UK Limited, Milton Keynes, United Kingdom) with the injection of 10 µL onto a Waters Acquity UPLC® CSH C18 column (Waters, Hertfordshire, United Kingdom); 1.7 µm, I.D. 2.1 mm X 50 mm, maintained at 55 degrees Celsius. Mobile phase A was 6:4, acetonitrile and water with 10 mM ammonium formate. Mobile phase B was 9:1, propan-2-ol and acetonitrile with 10 mM ammonium formate. The flow was maintained at 500 µL per minute through the following gradient: 0.00 minutes_40% mobile phase B; 0.40 minutes_43% mobile phase B; 0.45 minutes_50% mobile phase B; 2.40 minutes_54% mobile phase B; 2.45 minutes_70% mobile phase B; 7.00 minutes_99% mobile phase B; 8.00 minutes_99% mobile phase B; 8.3 minutes_40% mobile phase B; 10 minutes_40% mobile phase B. The sample injection needle was washed using 9:1, 2-propan-2-ol and acetonitrile. The mass spectrometer used was the Thermo Scientific Exactive Orbitrap with a heated electrospray ionization source (Thermo Fisher Scientific, Hemel Hempstead, UK). The mass spectrometer was calibrated immediately before sample analysis using positive and negative ionization calibration solution (recommended by Thermo Scientific). Additionally, the mass spectrometer scan rate was set at 4 Hz, giving a resolution of 25,000 (at 200 m/z) with a full-scan range of m/z 100 to 1,800 with continuous switching between positive and negative mode.

*Data processing—*The instrument responses of the analytes were normalized to the relevant internal standard response (producing area ratios), these area ratios corrected the intensity for any extraction and instrument variations. The area ratios were then blank corrected where intensities less than three times the blank samples were set to a ‘Not Found’ result (i.e., zero concentration). The accepted area ratios were then multiplied by the concentration of the internal standard to give the analyte semi-quantitative concentrations.

## Statistical analyses

Participants were initially separated into lean controls, obesity cohort, and biopsied NAFLD cases. Clinical and biochemical characteristics of obese controls and biopsied NAFLD cases were compared using two-way unpaired T-tests for continuous data and chi-squared test for categorical data. p-values were converted to q-values by adjusting for multiple testing using the Benjamini-Hochberg method.

Due to the differences in clinical characteristics and selection of participants, further analyses were performed within either the obesity cohort or biopsied NAFLD cases separately.

In the NAFLD cohort, the association between standard clinical or biochemical measurements and liver histology was tested using regression analysis. Skewed continuous independent variables (e.g. age, HOMA-IR), were logarithmically transformed and standardized (to mean = 0, standard deviation (SD) = 1) before being used in linear models. Univariable linear regression was performed for NAS, fibrosis, and portal inflammation score (dependent variables) and each independent variable.

Following initial processing as described above, only lipid species detected in >70% of participants (including lean controls) were included. For included species, minimum value imputation was used for missing values. ‘Total’ values for each lipid class (e.g. ‘total lysophosphatidylcholine (LPC)’) were calculated as the sum of all species within a class for each individual. Absolute concentrations of lipids were logarithmically transformed and standardized (to mean = 0, SD = 1) to account for skewed distributions and allow linear regression analyses.

In the obesity cohort, linear regression was performed between lipid species and serum alanine aminotransferase (ALT), HOMA-IR, hepatic fat fraction (HFF) on MRS, and liver stiffness (kPa) measured by transient elastography. In the biopsied NAFLD cases, linear regression was performed between lipid species and NAS, steatosis grade, portal inflammation score, fibrosis stage, and HOMA-IR. All linear regressions were adjusted for age and sex. Due to high correlation between lipid species, the critical p-value for significance was defined by 0.05/√n, where n is the number of included lipids: n=229 therefore p<3.3x10^-3^ was determined as statistically significant.

We then performed meta-regression to examine for trends in lipid saturation or carbon chain length within classes of lipids. Beta regression coefficients from the above models were regressed against double bonds or carbons within lipid classes. p-values were converted to q-values by adjusting for multiple testing using the Benjamini-Hochberg method.

## Comparison with adult NAFLD

We explored whether significant, directionally consistent associations were observed between lipids identified in this study and those in a previously published cohort study of adults^(6)^. This study used both targeted and untargeted metabolomics in a population-based study of middle-aged adults who had undergone abdominal ultrasound for identification of hepatic steatosis, in addition to comprehensive metabolic analysis. Further details of the Fenland Cohort are described elsewhere^(7)^. We searched for all directionally consistent and significant (q<0.1) associations for the 72 lipids that reached statistical significance from our analyses in pediatric biopsied NAFLD cases.

## Annotation with disease outcomes and GWAS loci

The 72 significant lipids from this study were also annotated with results from a recent metabolite-wide association study of non-communicable diseases in adults^(8)^. This study used untargeted metabolomics in the EPIC-Norfolk cohort of adults with long-term prospective follow-up for clinical outcomes, as a component of the EPIC study^(9)^. We searched for all directionally consistent and significant (q<0.1) associations from Pietzner *et al.* for the 72 lipids identified from our analyses in biopsied NAFLD cases.

Next, we explored which genetic variants are known to influence circulating levels of the lipids identified in the current analysis. We used data from a lipidomics genome-wide association study (GWAS), which had also collated relevant results from previous metabolomics GWAS^(10)^. We searched for genome-wide significant variants (as defined by the original studies) for the 72 top lipids identified in our analysis. The nearest gene annotation was extracted from the original study and no functional annotation of variants was performed.

Finally, for variants at GWAS significance from the above data, we performed a phenome-wide association study for cardio-metabolic traits. We used PheWAS data from Tabassum *et al.* and searched PhenoscannerV2^(11)^ to annotate variants.

All analyses were performed using R 4.0.2^(12)^ and code used is available from: https://doi.org/10.5281/zenodo.4656980.

**References**

1. van Werven JR, Marsman HA, Nederveen AJ, et al. Assessment of hepatic steatosis in patients undergoing liver resection: comparison of US, CT, T1-weighted dual-echo MR imaging, and point-resolved 1H MR spectroscopy. *Radiology* 2010; 256:159–168.

2. Vajro P, Lenta S, Socha P, et al. Diagnosis of nonalcoholic fatty liver disease in children and adolescents: position paper of the ESPGHAN Hepatology Committee. *J Pediatr Gastroenterol Nutr* 2012; 54:700–713.

3. Brunt EM, Kleiner DE, Wilson L a., et al. Portal chronic inflammation in nonalcoholic fatty liver disease (NAFLD): a histologic marker of advanced NAFLD-clinicopathologic correlation from the Nonalcoholic Steatohepatitis Clinical Research Network. *Hepatology* 2009; 49:809–820.

4. Kleiner DE, Brunt EM, Van Natta M, et al. Design and validation of a histological scoring system for nonalcoholic fatty liver disease. *Hepatology* 2005; 41:1313–1321.

5. Jenkins B, Ronis M, Koulman A. LC-MS Lipidomics: Exploiting a Simple High-Throughput Method for the Comprehensive Extraction of Lipids in a Ruminant Fat Dose-Response Study. *Metabolites* 2020; 10. https://doi.org/10.3390/metabo10070296

6. Mann JP, Pietzner M, Wittemans LB, et al. Insights into genetic variants associated with NASH-fibrosis from metabolite profiling. *Hum Mol Genet* 2020:doi: 10.1093/hmg/ddaa162.

7. Rolfe E, Loos RJ, Druet C, et al. Association between birth weight and visceral fat in adults. *Am J Clin Nutr* 2010; 92:347–352.

8. Pietzner M, Stewart ID, Raffler J, et al. Plasma metabolites to profile pathways in noncommunicable disease multimorbidity. *Nat Med* 2021; 27:471–479.

9. Day N, Oakes S, Luben R, et al. EPIC-Norfolk: Study design and characteristics of the cohort. *Br J Cancer* 1999; 80:95–103.

10. Tabassum R, Ramo JT, Ripatti P, et al. Genetic architecture of human plasma lipidome and its link to cardiovascular disease. *Nat Commun* 2019; 10:4329.

11. Kamat MA, Blackshaw JA, Young R, et al. PhenoScanner V2: an expanded tool for searching human genotype-phenotype associations. *Bioinformatics* 2019; 35:4851–4853.

12. R Core Team. A language and environment for statistical computing. Vienna, Austria: R Foundation for Statistical Computing. 2019.
