# Supplementary material for: Comparison of the lipidomic signature of fatty liver in children and adults: a cross-sectional study
Source: J Pediatr Gastroenterol Nutr. Author manuscript; Available in PMC 2022 Jul 8. (PMC7613028; doi:10.1097/MPG.0000000000003418)
Supplement: Supplemental digital content [file EMS143665-supplement-Supplemental_digital_content.docx]

**Supplemental digital content**

**Figure, Supplemental Digital Content 1. Overview of study design.** Three groups of a participants were included: lean controls, an obesity cohort, and cases biopsied for NAFLD. The association between baseline characteristics and histological severity of NAFLD was tested using linear regression. Plasma lipidomics was run in all three groups and analyzed using linear regression for HOMA-IR and liver-related traits. Meta-regression was performed on results from the obesity cohort and biopsied NAFLD cases. The 72 lipids associated with severity of NAFLD were annotated with four sets of results from adults. ALT, alanine aminotransferase; HFF, hepatic fat fraction; HOMA-IR, homeostatic model of insulin resistance; mWAS, metabolite-wide association study; NAS, NAFLD Activity Score; TE, transient elastography.

**Methods, Supplemental Digital Content 2.** Supplementary methods, including statistical analysis and comparison with adult cohorts.

**Table, Supplemental Digital Content 3. Baseline characteristics of participants included in the study.** Lean children (n=19) were those undergoing endoscopy who had no evidence of gastrointestinal pathology. Obesity cohort (n=146) comprised children who were overweight or obese referred and for clinical assessment. The biopsied NAFLD cases (n=122) were children with suspected severe paediatric NAFLD who underwent liver biopsy. q-values represent false-discovery rate correct p-values between obesity and NAFLD cohorts, using unpaired t-tests for continuous traits and chi-squared for sex. Data represents mean (standard deviation) for continuous traits and number (%) for categorical traits.

**Figure, Supplemental Digital Content 4. Characteristics of obesity cohort and cases biopsied for NAFLD.** Obesity cohort (n=146) blue) was collected from any child who was overweight or obese referred for clinical assessment. The NAFLD cases (n=122, pink) were children with suspected severe paediatric NAFLD who underwent liver biopsy. There is no data shown from the lean group. The two overlying histograms illustrate the distribution of traits for body mass index (BMI) z-score (A), homeostatic model of insulin resistance (HOMA-IR, B), serum triglycerides (C), serum total cholesterol (D), and serum alanine aminotransferase (ALT, E). A subset (n=95) children from the obesity cohort underwent magnetic resonance spectroscopy (MRS) for estimation of liver fat (F). q-values represent comparison of means using t-test derived using the Benjamini-Hochberg method where significance is q<.05.

**Table, Supplemental Digital Content 5. Summary statistics from all analyses.** Raw summary statistics from all analyses described. Table S1: baseline histology from children biopsied for NAFLD; Table S2: association between baseline traits (e.g. body mass index, age) and histological outcomes; Table S3: full results of all lipid-trait associations tested using linear regression; Table S4: full results of all meta-regression analyses; Table S5: list of 72 lipid significantly associated with the histological severity of NAFLD; Table S6: significant associations in adult cohorts^(18, 19)^ for the same 72 lipids identified in Table S5; Table S7: genetic loci associated with the 72 lipids from Table S5; Table S8: traits associated with the genetic loci from Table S7. See spreadsheet index for further details.

**Figure, Supplemental Digital Content 6. Associations between baseline characteristics and severity of peri-portal inflammation in children biopsied for NAFLD (n=122).** None of the baseline characteristics demonstrated an association with severity of portal inflammation. Associations were tested using linear regression. q-values were derived using the Benjamini-Hochberg method where significance is q<.05.

**Figure, Supplemental Digital Content 7. Summary of analysis of plasma lipid profiles in children with NAFLD.** After normalization, there was no difference between lean (n=19), obese (n=146), and NAFLD (n=122) groups on principal component analysis (A). Lipids from each class were correlated using hierarchical clustering (B) but there was no clear distinction between obese and NAFLD groups. On meta-regression (C), lipid saturation and carbon chain length were significantly associated with several traits in both the obese and NAFLD groups. The cell color in (C) represents the meta-regression beta coefficient and stars illustrate q-values (derived using the Benjamini-Hochberg method) where significance is q<.05. Pearson correlation co-efficient (and associated p-values) between all analysed traits in obesity cohort (D) and NAFLD biopsy cases (E).

**Figure, Supplemental Digital Content 8. Associations between phosphatidylcholine (PC) species and fatty liver.** (A) Total PC by BMI z-score. (B) Association between PC(36:1) and hepatic fat fraction in obese cohort. (C) Association between total PC and NAFLD Activity Score. Meta-regressions are panels D-I: each dot represents a different lipid species and those in red are lipids significantly (q<.05). Associations with ALT in the obese cohort (n=146) panels D & E. Associations with hepatic fat fraction on MRS in the obese cohort (n=95) panels F & G. Associations with NAFLD Activity Score in the biopsied patients (n=122) panels H & I. The line of best fit for meta-regression graphs (D-I) illustrates the overall trend between PC chain length (D, F, & H) or saturation (E, G, & I) and liver-related traits. The shaded area is the 95% confidence interval from the meta-regression analysis.

**Figure, Supplemental Digital Content 9. Associations between triglyceride (TG) species and fatty liver.** (A) Total TG by cohort. (B) Total TG by BMI (body mass index) z-score. (C) Association between TG(46:1) and serum alanine aminotransferase (ALT) in the obese cohort. Meta-regressions are panels D-I: each dot represents a different lipid species and those in red are lipids significantly (q<.05). Associations with ALT in the obese cohort (n=146) panels D & E. Associations with hepatic fat fraction on MRS in the obese cohort (n=95) panels F & G. Associations with NAFLD Activity Score in the biopsied patients (n=122) panels H & I. The line of best fit for meta-regression graphs (D-I) illustrates the overall trend between TG chain length (D, F, & H) or saturation (E, G, & I) and liver-related traits. The shaded area is the 95% confidence interval from the meta-regression analysis.

**Figure, Supplemental Digital Content 10. Additional significant associations between serum lipid species from LC-MS and fatty liver in children.** Total lyso-phosphatidylcholines (LPC) were highest in the NAFLD cases (A) and positively associated with NAFLD Activity Score (B), particularly for the saturated LPC (e.g. LPC(18:0), C). Whereas total phosphatidylglycerols (PG, D) and total gangliosides (E) were lower in obese children than controls and lowest in the NAFLD cases. Results from meta-regression are shown in F & I, where each dot represents a different lipid species and those in red are lipids significantly associated with the trait on linear regression. Phosphatidylinositol (PI) chain length (F) and saturation (I) were negatively associated with NAFLD Activity Score. Some lipids, such as PG(40:0) (G) and hexosylceramide (40:1) (H) demonstrated non-linear associations with NAFLD activity score. Panels A, D, & E show results from comparison of means (using t-tests) for total lipid abundance for each species. Panels B, C, G, & H show results from linear regression used log-transformed lipid concentrations adjusted for age and sex, and give p-values where significance is p<3.3x10^-3^. Panels F & I show results from meta-regression: the trend line is accompanied by a grey shaded area to illustrate the 95% confidence interval and q-values (derived using the Benjamini-Hochberg method) where significance is q<.05.
